# Severe and predominantly active atopic eczema in adulthood and long term risk of cardiovascular disease: population based cohort study

**DOI:** 10.1136/bmj.k1786

**Published:** 2018-05-23

**Authors:** Richard J Silverwood, Harriet J Forbes, Katrina Abuabara, Anna Ascott, Morten Schmidt, Sigrún A J Schmidt, Liam Smeeth, Sinéad M Langan

**Affiliations:** 1 Faculty of Epidemiology and Population Health, London School of Hygiene and Tropical Medicine, London WC1E 7HT, UK; 2 Program for Clinical Research, Department of Dermatology, University of California, San Francisco School of Medicine, San Francisco, CA, USA; 3 Royal Sussex County Hospital, Brighton, UK; 4 Department of Clinical Epidemiology, Aarhus University Hospital, Aarhus, Denmark; 5 Department of Cardiology, Regional Hospital West Jutland, Herning, Denmark

## Abstract

**Objective:**

To investigate whether adults with atopic eczema are at an increased risk of cardiovascular disease and whether the risk varies by atopic eczema severity and condition activity over time.

**Design:**

Population based matched cohort study.

**Setting:**

UK electronic health records from the Clinical Practice Research Datalink, Hospital Episode Statistics, and data from the Office for National Statistics, 1998–2015.

**Participants:**

Adults with a diagnosis of atopic eczema, matched (on age, sex, general practice, and calendar time) to up to five patients without atopic eczema.

**Main outcome measures:**

Cardiovascular outcomes (myocardial infarction, unstable angina, heart failure, atrial fibrillation, stroke, and cardiovascular death).

**Results:**

387 439 patients with atopic eczema were matched to 1 528 477 patients without atopic eczema. The median age was 43 at cohort entry and 66% were female. Median follow-up was 5.1 years. Evidence of a 10% to 20% increased hazard for the non-fatal primary outcomes for patients with atopic eczema was found by using Cox regression stratified by matched set. There was a strong dose-response relation with severity of atopic eczema. Patients with severe atopic eczema had a 20% increase in the risk of stroke (hazard ratio 1.22, 99% confidence interval 1.01 to 1.48), 40% to 50% increase in the risk of myocardial infarction, unstable angina, atrial fibrillation, and cardiovascular death, and 70% increase in the risk of heart failure (hazard ratio 1.69, 99% confidence interval 1.38 to 2.06). Patients with the most active atopic eczema (active >50% of follow-up) were also at a greater risk of cardiovascular outcomes. Additional adjustment for cardiovascular risk factors as potential mediators partially attenuated the point estimates, though associations persisted for severe atopic eczema.

**Conclusions:**

Severe and predominantly active atopic eczema are associated with an increased risk of cardiovascular outcomes. Targeting cardiovascular disease prevention strategies among these patients should be considered.

## Introduction

Atopic eczema affects up to 10% of adults and is becoming more common worldwide.^1^ It is caused by both skin barrier and immune system defects, and there is increasing evidence that the systemic inflammatory component of atopic eczema may contribute to other conditions, including cardiovascular outcomes.^2^ Given the prevalence of atopic eczema, even a small increase in cardiovascular risk would be important from a public health perspective.

Mixed findings have been reported in cohort studies assessing associations between atopic eczema and acute cardiovascular outcomes from Taiwan, Denmark, and the USA.^3^ ^4^ ^5^ It is unclear if any increased risk is explained by increased cardiovascular risk factors in patients with atopic eczema.^3^ In studies assessing the association between atopic eczema and acute cardiovascular outcomes where temporality can be inferred, key data on important lifestyle and anthropometric confounders, such as body mass index and smoking, are largely unavailable. Atopic eczema is a relapsing and remitting condition which varies in severity and eczema activity, and it is also unclear whether the potential association differs by these characteristics. Estimates of the spectrum of atopic eczema severity vary depending on the approach used to define severity.^6^ Previous reports suggest that around 30% of patients with atopic eczema would be classified as having moderate or severe atopic eczema.^7^ ^8^ ^9^

We therefore examined whether adults with atopic eczema are at a greater risk of cardiovascular events. We also investigated whether the risk of cardiovascular disease varied by atopic eczema severity and eczema activity over time.

## Methods

### Study design and setting

We undertook a cohort study with data from the UK Clinical Practice Research Datalink (CPRD), linked to Hospital Episode Statistics inpatient data, Office for National Statistics mortality data, and index of multiple deprivation data. CPRD is a database of prospectively collected primary care records from general practitioners using Vision software; approximately 9% of the UK population are represented in the database.^10^ ^11^ Data are anonymised and include diagnoses (coded using Read codes), prescriptions (coded using British National Formulary codes), and referrals to specialists. Approximately 80% of CPRD practices registered in England have consented to their patients’ primary care records being linked to other data sources. Hospital Episode Statistics include all NHS inpatient hospital stays in England since 1997, with diagnoses coded using ICD-10 (international classification of diseases, 10th revision) codes and procedures coded according to OPCS Classification of Interventions and Procedures codes. Office for National Statistics linked mortality data contain the underlying cause of death, recorded on the death certificate, along with up to 15 other recorded causes of death. Causes are coded using ICD-9 codes before January 2001 and ICD-10 codes thereafter. Index of multiple deprivation data contain quintiles of deprivation based on the patient’s postcode. For this study, data were extracted from the January 2016 CPRD build and the Set 12 linked data. 

### Study population

Adult patients (aged ≥18) contributing to CPRD between 2 January 1998 and 31 March 2015, with linked Hospital Episode Statistics data were eligible for inclusion in the study.

#### Defining patients with atopic eczema 

The exposed cohort included all patients with atopic eczema. We defined atopic eczema onset as the latest of an atopic eczema diagnosis and two atopic eczema treatments (on separate dates), consistent with a validation study showing a positive predictive value in adults of 82% (95% confidence interval 73% to 89%).^12^ Atopic eczema diagnostic codes were identified in CPRD (using Read codes) and Hospital Episode Statistics (using ICD-10 codes in the primary diagnosis field of any episode). Treatments included atopic eczema related prescriptions from primary care: emollients, topical and oral corticosteroids, tacrolimus and systemic immunosuppressants (methotrexate, ciclosporin, mycophenolate mofetil, or azathioprine), and phototherapy records from CPRD or OPCS Classification of Interventions and Procedures codes in Hospital Episode Statistics.

#### Defining matched unexposed patients

For each patient with atopic eczema, we randomly matched up to five patients by age (within 15 years), sex, general practice, and calendar time at cohort entry. These unexposed patients were required to have at least one year of follow-up in CPRD and no history of atopic eczema when matched. Any patients with a diagnosis of atopic eczema were included in the pool of eligible unexposed patients until the date of their diagnosis of atopic eczema; before their diagnosis of atopic eczema, these patients were not considered to have atopic eczema and were therefore eligible to contribute to unexposed person time. Patients with a diagnosis of atopic eczema who did not go on to meet the full definition of atopic eczema (at least one diagnosis code and two treatment codes on separate dates) were also in the pool of eligible unexposed patients up until the date of their atopic eczema diagnosis code. Removing these patients from the pool of eligible unexposed patients at the point of diagnosis rather than allowing them to remain until they met the full validated definition of atopic eczema ensured greater certainty that the pool of unexposed patients did not have atopic eczema. Patients could only be sampled as unexposed once during the study (ie, sampling without replacement).

#### Exclusions

After matching, we excluded patients with codes indicating previous or current cardiovascular disease, including all the main outcome codes plus further codes including history (or possible history) of each outcome, epidural or subdural strokes, subarachnoid haemorrhagic strokes, and risk factors for subarachnoid haemorrhage, such as cerebral aneurysms in the circle of Willis or arteriovenous malformations.

### Defining follow-up

Follow-up for exposed patients began at the latest of: 2 January 1998, the date the individual turned 18 years old, the date of diagnosis of atopic eczema, or the start of CPRD follow-up plus 365 days (to ensure a cardiovascular outcome diagnosis was incident and not recorded retrospectively after registration at a general practice).^13^ Follow-up for unexposed individuals began at the start date of their matched patient with atopic eczema. Follow-up ended at the earliest of study end date, death (using Office for National Statistics date or, if missing, CPRD death date), transfer out of practice, practice last collection date, or the patient developing an outcome of interest. For analyses concerning stroke only, follow-up ended on the earliest of study end date, death, transfer out of practice, practice last collection date, the patient developing an outcome of interest, or the date of an epidural or subdural stroke, subarachnoid haemorrhagic stroke, or risk factor for subarachnoid haemorrhage (such as embolisation of cerebral artery and non-ruptured cerebral aneurysm). Patients contributing at least one day of follow-up were included in the study.

### Severe and predominantly active atopic eczema

Severity of atopic eczema was defined as a time-updated variable for patients with atopic eczema. Patients with atopic eczema were considered to have mild conditions by default. They were classified as having moderate atopic eczema at the first of: a second potent topical corticosteroid treatment within one year or a first calcineurin inhibitor treatment. Patients were classified as having severe atopic eczema at the first of: a systemic immunosuppressant treatment; a phototherapy code in CPRD or Hospital Episode Statistics; or a referral for atopic eczema. Once defined as moderate, patients with atopic eczema remained as such unless they developed severe atopic eczema; once defined as severe, patients with atopic eczema remained as such, similar to established approaches for defining severity in psoriasis studies.^14^ At any given point during follow-up, patients with atopic eczema therefore belonged to one of three severity categories: mild, moderate, or severe.

Atopic eczema activity was assessed over time. Active atopic eczema started at the latest of two CPRD or Hospital Episode Statistics atopic eczema records (either diagnoses or treatment) appearing within any one year period. Active atopic eczema was assumed to last for 12 months, unless another atopic eczema record appeared, in which case its duration was prolonged for another 12 months. Patients with atopic eczema were subsequently split into three categories for analysis: those who never had active atopic eczema during follow-up, those who had active atopic eczema for less than 50% of follow-up, and those who had active atopic eczema for at least 50% of follow-up.

### Cardiovascular outcomes

We identified the following individual cardiovascular diseases in CPRD and Hospital Episode Statistics (primary diagnosis fields of any episode): myocardial infarction, unstable angina, heart failure, atrial fibrillation, stroke (ischaemic, haemorrhagic, or unspecified), and cardiovascular death. Secondary outcomes included coronary revascularisation procedures, identified in OPCS Classification of Interventions and Procedures data. Deaths from cardiovascular disease were identified from Office for National Statistics data, defined as any ICD-9 or ICD-10 code related to cardiovascular disease recorded as a cause of death.

### Covariates

We used a directed acyclic graph to inform the identification of covariates and mediators and to avoid collider bias (see supplementary figure S1).^15^ The covariates included current age, calendar period (1997-99, 2000-04, 2005-09, 2010-15), time since diagnosis (0-4, 5-9, 10-14, 15-19, ≥20 years), and socioeconomic status (quintiles of 2015 index of multiple deprivation). Patients were defined as having diabetes mellitus (type 1, type 2, or missing), hypertension, hyperlipidaemia, depression, anxiety, or asthma on the date of their first ever Read code in CPRD for these conditions, which may have been after cohort entry, as a time updated variable. Body mass index (according to WHO categories) and smoking status (current smoker, former smoker, or non-smoker) were defined at cohort entry. Patients were defined as having severe alcohol use at the first of: high alcohol use code or treatment for severe alcohol use recorded in CPRD. Patients were defined as exposed to high dose (≥20 mg/day) oral corticosteroids for the duration of their prescription and three months after the end of the prescription. Further details regarding the definition of these variables can be found in the supplementary material.

### Statistical analysis

#### Primary analyses

The characteristics of those with and without atopic eczema (at cohort entry) were described. We used Cox regression stratified by matched set (matched on age at cohort entry, sex, general practice, and date at cohort entry) with current age as the underlying timescale to generate hazard ratios for the association between atopic eczema and each cardiovascular outcome (the unadjusted model). Subsequent multivariable analyses adjusted for current calendar period (1997-99, 2000-04, 2005-09, 2010-15), current time since diagnosis (0-4, 5-9, 10-14, 15-19, ≥20 years), socioeconomic status, and time varying asthma (the adjusted model). The adjusted model was further adjusted for variables which may have been on the causal pathway (ie, mediators) between atopic eczema and cardiovascular outcomes (smoking and body mass index at cohort entry, and time varying diabetes, hypertension, hyperlipidaemia, depression and anxiety, and severe alcohol use) (the mediation model). Patients with missing body mass index, smoking, or index of multiple deprivation data were excluded. These data are likely to be missing not at random (as missingness is likely to depend on the actual values). Multiple imputation would therefore not be appropriate. Complete case analysis is valid where the missingness is independent of each of the cardiovascular outcomes, conditional on the model covariates.^16^ We used 99% confidence intervals and an implied 1% level of statistical significance to reduce the risk of type 1 error.

Incidence rates of each cardiovascular outcome in the patients with atopic eczema were estimated by using the data in our sample. Incidence rates of each cardiovascular outcome among patients without atopic eczema (which cannot be reliably estimated from the sample owing to the matching) were then estimated by multiplying the incidence rate in the patients with atopic eczema by our corresponding estimated hazard ratio (after having first inverted it so that it compares unexposed with exposed). We calculated attributable risks as the difference between these exposure group specific incidence rates. The population attributable risk of each cardiovascular outcome was estimated by using the estimated hazard ratio and assuming the prevalence of atopic eczema to be 10%.^17^

#### Secondary analyses

We repeated the analyses within strata of sex, current asthma status, and current age (18-39, 40-59, and ≥60) to explore potential effect modification. We also repeated the analysis with alternative exposure definitions, where atopic eczema was categorised based on severity and, separately, on category of eczema activity.

#### Sensitivity analyses and model checking

Table 1 shows the series of sensitivity analyses we conducted. We also checked the proportional hazards assumption in all the primary analysis models through plots of the Schöenfeld residuals.

**Table 1:** Sensitivity analyses

Sensitivity analysis	Justification
The activity analysis was repeated, restricted to patients with at least five years of follow-up	To explore any potential bias caused by patients with atopic eczema with short follow-up periods being more likely to have either none or all of their follow-up with active atopic eczema
The primary analysis was repeated on an incident atopic eczema cohort (exposed patients defined as those joining the cohort when they first fulfil our diagnostic criteria and after the start of the study period)	Covariates measured at entry precede atopic eczema onset so will not be on the causal pathway between atopic eczema and cardiovascular outcomes
The primary analysis was repeated on patients with at least one consultation with their doctor in the year before cohort entry	To exclude practice non-attenders
The primary analysis was repeated on a redefined cohort, where the pool of unexposed patients also included patients with an atopic eczema diagnosis but without two further treatments for the entire duration of their follow-up and patients in the exposed cohort (with an atopic eczema diagnosis and two further treatments) were included as unexposed up until their cohort entry (ie, the latest of their atopic eczema diagnosis and their two further treatments)	To explore the sensitivity of the results to the definition of the exposure
The primary analysis was repeated on a second redefined cohort where exposed patients were those with an atopic eczema diagnosis only (ie, without requiring two atopic eczema treatments), and these patients were eligible for the unexposed cohort up until their atopic eczema diagnosis (some patients may have childhood atopic eczema, but may not have treatment codes recorded if they registered at the practice during adulthood, and therefore may be erroneously excluded from the exposed cohort in the primary analysis)	To explore the sensitivity of the results to the definition of the exposure
The primary analysis was repeated on a subset of patients registered from 2007 onwards	Data on covariates, particularly body mass index and smoking, would be expected to be more complete, thus reducing any selection bias owing to missing data
The primary analysis was repeated on a subset of patients registered from 2007 onwards, additionally adjusting for ethnic group (white, South Asian, black, other, or mixed, identified from Clinical Practice Research Datalink and Hospital Episode Statistics data using a previously developed algorithm)^18^	To examine whether the omission of this covariate in the primary analysis may have introduced bias
The primary analysis (mediation model only) was repeated with additional adjustment for time updated use of high dose corticosteroids	To examine whether the omission of this covariate in the primary analysis may have introduced bias

### Patient involvement

The research questions, design, conduct, and initial results and interpretation of the findings of this study have been overseen by the Wellcome Senior Clinical Fellowship steering committee, which includes lay representation. No patients were asked to advise on interpretation or writing up of results. There are no plans to disseminate the results of the research to study participants.

## Results

**Fig 1:**
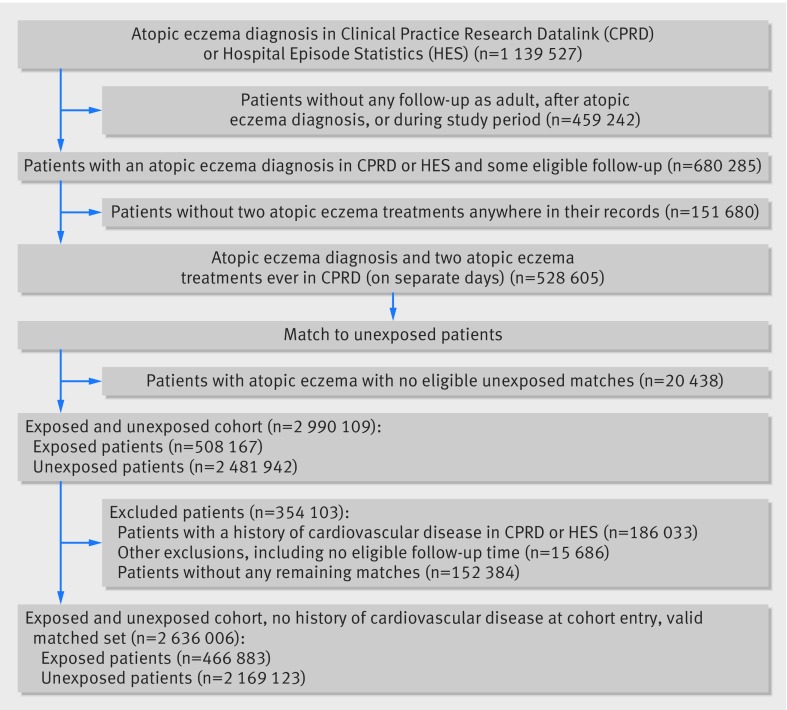
Flowchart for cohort study, 1998-2015

Figure 1 shows that in total, 466 883 exposed and 2 169 123 unexposed patients were successfully matched and were eligible for cohort entry. Of these, 517 444 (19.6%) were excluded from subsequent analyses as they did not have complete data on the analysis variables (19.2% missing body mass index, 5.3% missing smoking, and 0.1% missing index of multiple deprivation), and a further 202 646 (7.7%) patients were excluded as they had no remaining matches. This left a final analysis sample of 387 439 exposed and 1 528 477 unexposed patients. Table 2 and supplementary table S1 show that distributions of variables did not differ substantially between those included in the final analysis sample and the overall sample, except for a somewhat higher proportion of exposed patients (20.2% *v* 17.7%), more female patients (66.0% *v* 60.0%), and fewer of the very youngest (aged 18–19) patients (6.5% *v* 14.5%) in the analysis sample. Median follow-up was 5.1 years and over the follow-up period, 19 700 (5.1%) patients with atopic eczema experienced severe atopic eczema.

**Table 2:** Covariate summary statistics for patients with complete data on all analysis variables and belonging to valid matched sets.* Values are mean (percentage) unless stated otherwise

Characteristic	Without atopic eczema (n=1 528 477) (79.8%))	With atopic eczema (n=387 439 (20.2%))	Total (n=1 915 916)
**Follow**-**up (years)**			
Median (interquartile range)	4.9 (2.0-9.6)	5.7 (2.4-10.3)	5.1 (2.0-9.8)
**At entry to cohort**			
Sex:			
Male	511 676 (33.5)	139 908 (36.1)	651 584 (34.0)
Female	1 016 801 (66.5)	247 531 (63.9)	1 264 332 (66.0)
Age (years):			
18-19	87 600 (5.7)	36 392 (9.4)	123 992 (6.5)
20-29	275 632 (18.0)	66 156 (17.1)	341 788 (17.8)
30-39	315 206 (20.6)	70 222 (18.1)	385 428 (20.1)
40-49	272 436 (17.8)	61 898 (16.0)	334 334 (17.5)
50-59	243 469 (15.9)	55 294 (14.3)	298 763 (15.6)
60-69	195 936 (12.8)	49 420 (12.8)	245 356 (12.8)
70-79	104 132 (6.8)	33 837 (8.7)	137 969 (7.2)
≥80	34 066 (2.2)	14 220 (3.7)	48 286 (2.5)
Index of multiple deprivation:			
1 (least deprived)	370 953 (24.3)	94 223 (24.3)	465 176 (24.3)
2	332 853 (21.8)	84 297 (21.8)	417 150 (21.8)
3	312 836 (20.5)	78 603 (20.3)	391 439 (20.4)
4	273 332 (17.9)	69 223 (17.9)	342 555 (17.9)
5 (most deprived)	238 503 (15.6)	61 093 (15.8)	299 596 (15.6)
Body mass index:			
Underweight	45 488 (3.0)	11 590 (3.0)	57 078 (3.0)
Normal weight	709 903 (46.4)	174 234 (45.0)	884 137 (46.1)
Overweight	484 434 (31.7)	123 352 (31.8)	607 786 (31.7)
Obese	288 652 (18.9)	78 263 (20.2)	366 915 (19.2)
Smoking status:			
Non	699 570 (45.8)	168 221 (43.4)	867 791 (45.3)
Current	480 780 (31.5)	116 551 (30.1)	597 331 (31.2)
Former	348 127 (22.8)	102 667 (26.5)	450 794 (23.5)
Diabetes	51 213 (3.4)	15 777 (4.1)	66 990 (3.5)
Hypertension	190 217 (12.4)	58 001 (15.0)	248 218 (13.0)
Hyperlipidaemia	59 376 (3.9)	19 342 (5.0)	78 718 (4.1)
Depression	300 699 (19.7)	95 131 (24.6)	395 830 (20.7)
Anxiety	194 289 (12.7)	65 248 (16.8)	259 537 (13.5)
Asthma	190 728 (12.5)	91 955 (23.7)	282 683 (14.8)
Severe alcohol use	28 803 (1.9)	8730 (2.3)	37 533 (2.0)

* Matched sets including one exposed patient and at least one unexposed patient.

Table 3 shows that in the primary analysis, there was evidence of associations between atopic eczema and all cardiovascular outcomes, except for cardiovascular death. Associations were strongest with unstable angina (hazard ratio 1.25, 99% confidence interval 1.11 to 1.41 in the adjusted model) and heart failure (1.19, 1.10 to 1.30), with partial attenuation in the mediation model.

**Table 3:** Association between atopic eczema and cardiovascular outcomes. Fitted to patients with complete data for all variables included in the models and from valid matched sets* (n=1  915  916, 1  842  759 unique patients)

Variables	No	Patient years at risk	Events		Hazard ratio (99% CI)†		
					Unadjusted	Adjusted‡	Mediation model§
**Primary outcomes**							
Myocardial infarction:							
Unexposed	1 528 477	9 361 522	17 178		1.00 (ref)	1.00 (ref)	1.00 (ref)
Exposed	387 439	2 569 214	5561		1.10 (1.05 to 1.15)	1.06 (0.98 to 1.15)	1.04 (0.96 to 1.13)
Unstable angina:							
Unexposed	1 528 477	9 392 370	7059		1.00 (ref)	1.00 (ref)	1.00 (ref)
Exposed	387 439	2 578 165	2460		1.22 (1.14 to 1.31)	1.25 (1.11 to 1.41)	1.17 (1.03 to 1.32)
Heart failure:							
Unexposed	1 528 477	9 375 383	16 983		1.00 (ref)	1.00 (ref)	1.00 (ref)
Exposed	387 439	2 570 412	6441		1.21 (1.16 to 1.26)	1.19 (1.10 to 1.30)	1.17 (1.08 to 1.28)
Atrial fibrillation:							
Unexposed	1 528 477	9 316 331	28 571		1.00 (ref)	1.00 (ref)	1.00 (ref)
Exposed	387 439	2 552 311	9892		1.12 (1.08 to 1.16)	1.11 (1.04 to 1.18)	1.07 (1.00 to 1.15)
Stroke:							
Unexposed	1 528 477	9 361 252	21 387		1.00 (ref)	1.00 (ref)	1.00 (ref)
Exposed	387 439	2 568 749	7149		1.07 (1.03 to 1.12)	1.10 (1.02 to 1.19)	1.08 (1.00 to 1.16)
Cardiovascular death:							
Unexposed	1 528 477	9 427 420	30 116		1.00 (ref)	1.00 (ref)	1.00 (ref)
Exposed	387 439	2 590 305	10 813		1.07 (1.03 to 1.11)	0.98 (0.92 to 1.06)	0.96 (0.89 to 1.03)
**Secondary outcome**							
Coronary revascularisation:							
Unexposed	1 528 477	9 358 381	16 195		1.00 (ref)	1.00 (ref)	1.00 (ref)
Exposed	387 439	2 567 932	5056		1.12 (1.07 to 1.17)	1.14 (1.05 to 1.24)	1.08 (0.99 to 1.17)

* Matched sets including one exposed patient and at least one unexposed patient.

† Estimated hazard ratios from Cox regression with current age as underlying timescale, stratified by matched set (matched on age at cohort entry, sex, general practice, and date at cohort entry).

‡ Adjusted for current calendar period (1997-99, 2000-04, 2005-09, 2010-15), time since diagnosis (0-4, 5-9, 10-14, 15-19, ≥20 years), index of multiple deprivation at cohort entry, and time-varying asthma.

§ Adjusted additionally for body mass index and smoking at cohort entry, and time-varying hyperlipidaemia, hypertension, depression, anxiety, diabetes, and severe alcohol use.

Table 4 shows that the estimated attributable risks confirm the increased incidence rates of cardiovascular outcomes among patients with atopic eczema. Attributable risks were greatest for heart failure (40 per 100 000, 99% confidence interval 22 to 57) and atrial fibrillation (37, 15 to 55). The greatest population attributable risks were estimated for unstable angina (2.4%, 1.1% to 3.9%) and heart failure (1.9%, 1.0% to 2.9%).

**Table 4:** Absolute incidence rates, incidence rate differences (attributable risks), and population attributable risks of cardiovascular outcomes

Variables	Estimated incidence rate* in patients with atopic eczema	Hazard ratio (99% CI)†	Inverse hazard ratio (99% CI)‡	Estimated incidence rate* (99% CI) in patients without atopic eczema	Estimated incidence rate difference* (99% CI)	Estimated population attributable risk (%) (99% CI)§
**Primary outcomes**						
Myocardial infarction	205	1.06 (0.98 to 1.15)	0.94 (0.87 to 1.02)	193 (178 to 209)	12 (−4 to 27)	0.6 (−0.2 to 1.5)
Unstable angina	89	1.25 (1.11 to 1.41)	0.80 (0.71 to 0.90)	71 (63 to 80)	18 (9 to 26)	2.4 (1.1 to 3.9)
Heart failure	248	1.19 (1.10 to 1.30)	0.84 (0.77 to 0.91)	208 (191 to 226)	40 (22 to 57)	1.9 (1.0 to 2.9)
Atrial fibrillation	366	1.11 (1.04 to 1.18)	0.90 (0.85 to 0.96)	329 (311 to 351)	37 (15 to 55)	1.1 (0.4 to 1.8)
Stroke	276	1.10 (1.02 to 1.19)	0.91 (0.84 to 0.98)	251 (232 to 270)	25 (6 to 44)	1.0 (0.2 to 1.9)
Cardiovascular death	440	0.98 (0.92 to 1.06)	1.02 (0.94 to 1.09)	449 (414 to 480)	−9 (−40 to 26)	−0.2 (−0.8 to 0.6)
**Secondary outcome**						
Coronary revascularisation	179	1.14 (1.05 to 1.24)	0.88 (0.81 to 0.95)	158 (145 to 170)	21 (9 to 34)	1.4 (0.5 to 2.3)

* Per 100 000 person years

† Adjusted for current calendar period (1997-99, 2000-04, 2005-09, 2010-15), time since diagnosis (0-4, 5-9, 10-14, 15-19, ≥20 years), index of multiple deprivation at cohort entry, and time-varying asthma.

‡ Comparing patients without atopic eczema to patients with atopic eczema.

§ Estimated as P(HR-1)**/**(1+P(HR-1)) where P, the prevalence of atopic eczema, is assumed to be 10% and HR is the estimated hazard ratio‡.^17^

There was no convincing evidence of effect modification by age, sex, or current asthma status (see supplementary tables S2-4).

**Fig 2:**
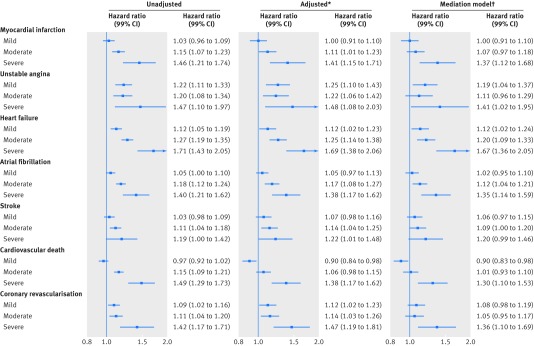
Association between atopic eczema and cardiovascular outcomes, by severity of atopic eczema versus no eczema. *Adjusted for current calendar period (1997-99, 2000-04, 2005-09, 2010-15), time since diagnosis (0-4, 5-9, 10-14, 15-19, ≥20 years), index of multiple deprivation at cohort entry, and time-varying asthma. †Adjusted additionally for body mass index and smoking at cohort entry, and time-varying hyperlipidaemia, hypertension, depression, anxiety, diabetes, and severe alcohol use

**Fig 3:**
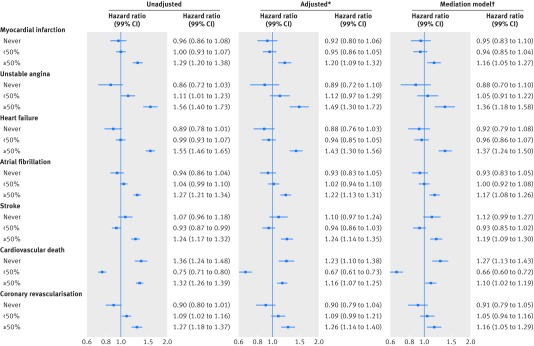
Association between atopic eczema and cardiovascular outcomes, by activity of atopic eczema versus no eczema. *Adjusted for current calendar period (1997-99, 2000-04, 2005-09, 2010-15), time since diagnosis (0-4, 5-9, 10-14, 15-19, ≥20 years), index of multiple deprivation at cohort entry, and time-varying asthma. †Adjusted additionally for body mass index and smoking at cohort entry, and time-varying hyperlipidaemia, hypertension, depression, anxiety, diabetes, and severe alcohol use

Effect estimates were substantially stronger in patients with severe atopic eczema, in particular for unstable angina (hazard ratio 1.48, 99% confidence interval 1.08 to 2.03 in the adjusted model) and heart failure (1.69, 1.38 to 2.06) (see fig 2 and supplementary table S5). Similar findings were observed for patients with the most active atopic eczema, in particular for unstable angina (1.49, 1.30 to 1.72) and heart failure (1.43, 1.30 to 1.56) (see fig 3 and supplementary table S6).

### Sensitivity analyses

Restricting the activity analysis to patients with at least five years of follow-up (43% of all patients) generally gave similar findings, though results for cardiovascular death changed more markedly, with the estimates for the never and <50% activity groups attenuated towards the null (see supplementary table S7). Results from the analysis with the incident atopic eczema cohort (54% of those in the primary analysis) were very similar to those in the primary analysis (see supplementary table S8). When analyses were restricted to patients with at least one consultation with their doctor in the year before cohort entry (83% of all patients) the estimated hazard ratios were attenuated slightly relative to those in the primary analysis (see supplementary table S9). The results from the analyses with the two redefined patient cohorts differed somewhat from those in the primary analysis (see supplementary tables S10 and S11); however, all confidence intervals included the point estimate from the main analysis. Only 11% of patients had registrations from 2007 onwards, and this small subset of patients differed substantially from the primary analysis sample, particularly in terms of age (younger; see supplementary table S12), and therefore the hazard ratios differed markedly (see supplementary table S13). Results adjusted for ethnic group (restricting to the 9% of patients with such data) were consistent with the main analysis (see supplementary table S14), suggesting limited bias from omission of this covariate. Results adjusted for time updated use of high dose corticosteroids were consistent with the main analysis (see supplementary table S15), similarly suggesting limited bias from omission of this covariate.

## Discussion

This study shows that atopic eczema is associated with a moderately increased risk of non-fatal cardiovascular outcomes, with a dose-response for atopic eczema severity and cumulated activity. Patients with severe atopic eczema were at a 20% increased risk of stroke, 40-50% increased risk of unstable angina, myocardial infarction, atrial fibrillation, and cardiovascular death, and 70% increased risk of heart failure. The most active atopic eczema group had similar findings. Estimated attributable risks confirm the increased incidence rates of cardiovascular outcomes among patients with atopic eczema, with population attributable risks of 2% or more for some outcomes.

### Comparison with other studies

Mechanistic work suggests that atopic eczema may be associated with increased platelet activation and decreased fibrinolysis, which may increase the risk of clotting,^19^ though a recent study found no association with metabolite levels.^20^ The association between atopic eczema and cardiovascular outcomes has been inconsistent in the literature. Studies from Taiwanese populations report a 1.33-fold increased incidence of stroke, rising to 1.71-fold increase in those with severe atopic eczema.^4^ Studies in Danish and German populations have reported positive associations between severe atopic eczema and cardiovascular outcomes (including angina, myocardial infarction, and stroke), whereas the association with mild atopic eczema has been either slightly protective or close to zero.^3^ ^20^ This may suggest a dose-response effect, an alternative pathogenesis underlying mild compared with severe conditions, the effect of systemic therapies used to treat severe forms of atopic eczema, or misclassification bias owing to the way in which patients with atopic eczema were classified. The European and Taiwanese studies used data from administrative databases which are likely to have incomplete data on potentially important cardiovascular risk factors including body mass index, smoking, and alcohol consumption. These factors may be more prevalent in patients with atopic eczema and could therefore contribute to an increased risk of cardiovascular outcomes.^21^ Our cohort study is one of the few longitudinal studies to have adjusted for these risk factors. Silverberg et al adjusted for body mass index, smoking, alcohol consumption, and physical activity in three cross sectional surveys in USA populations, and still found strong associations between atopic eczema and angina, coronary artery disease, myocardial infarction, and stroke.^22^ However, these studies were cross sectional, and misclassification is likely owing to exposures and outcomes being self reported. A study in a specific USA population found no noticeable association between self reported atopic eczema and either myocardial infarction or stroke in female nurses.^5^

### Strengths and weaknesses of this study

This is the largest study to assess the association between atopic eczema and major cardiovascular events. It is also the first study based on primary care data, meaning that our results are generalisable to a broad population with atopic eczema. We used population based data from UK general practices with linked data on hospital stays and cause specific mortality.^23^ ^24^ Previous studies have shown that this population is largely representative of the general UK population.^11^ We used a validated algorithm to identify atopic eczema and our approach to defining atopic eczema severity showed a similar distribution of severity to the literature.^12^ ^14^ For most of our study population, we had data on body mass index, smoking, and severe alcohol use, allowing us to adjust for potential mediators between atopic eczema and cardiovascular outcomes. We used a directed acyclic graph to identify covariates and mediators, and to avoid collider bias.^15^ The study outcomes require medical intervention and therefore presentation in primary or secondary care, meaning that ascertainment bias is unlikely to be a problem. One exception may be asymptomatic atrial fibrillation, though this is only a minor proportion of the total patients with atrial fibrillation so any ascertainment bias is likely to be limited.

Limitations of the study, inherent to most large observational studies, include the possibility for confounding, bias, and missing data. It is not possible to separate the effects of therapy and severity, owing to the nature of routinely collected data sources and observational settings whereby those with more severe atopic eczema are given specific treatments, and where those treatments might be continued for long periods. Patients were matched within 15 year age intervals as finer age matching led to a noticeable loss of patients with atopic eczema, who did not have any eligible matches. However, age was accounted for as the underlying timescale in all analyses, closely adjusting for the confounding effects of age. Exclusion of patients without complete data on body mass index, smoking, and index of multiple deprivation reduced the sample by nearly 20%. However, we believe that there are no factors leading to missing data which would independently affect the cardiovascular outcomes in question, therefore using a complete case analysis was valid. Findings for cardiovascular death were counterintuitive, as low atopic eczema activity seemed protective against mortality relative to no atopic eczema. This finding may be explained by the poor capture of data on activity: some patients may have active atopic eczema but do not adhere to treatment, thus being misclassified as never active. As this association was largely attenuated when the analysis was restricted to patients with at least five years of follow-up, another possible explanation could be survival bias, but this needs to be confirmed in other studies. Assessing major long term health outcomes in a condition such as atopic eczema that frequently starts in early childhood may be challenging owing to possible survival bias; the prevalent cohort would have fewer patients rapidly developing the cardiovascular outcomes (those at greatest risk) as they may not be eligible for sampling.^25^ ^26^ As survival bias would bias the association between atopic eczema and cardiovascular disease towards the null, it cannot explain our findings, and could potentially have led to underestimation of the associations.^27^ Similarly to activity of atopic eczema, misclassification of condition severity is possible, for example if patients with severe conditions used only topical treatment. However, such misclassification would bias our result towards the null, underestimating the magnitude of the associations.

By contrast, bias owing to onset confounding (differential time since atopic eczema onset) may bias away from the null if the risk of cardiovascular disease increases with time since atopic eczema onset and most patients with atopic eczema are prevalent cases who may have a greater risk of cardiovascular disease. Consistency in results when restricting to incident atopic eczema diagnoses suggests that this is not a major issue.

### Conclusions

We have shown a clinically relevant increase in the risk of acute cardiovascular outcomes in patients with atopic eczema. This increased risk is largely confined to patients with severe or more active atopic eczema and persists despite adjusting for potential mediators, including conventional risk factors for cardiovascular outcomes. Consideration should be given to developing prevention strategies to reduce the risk of cardiovascular disease among patients with severe or predominantly active atopic eczema, including awareness of and screening for conventional cardiovascular risk factors by those providing clinical care. Current biological treatments for atopic eczema have the potential to greatly change care for those with challenging eczema. The next objective will be to reduce the risk of cardiovascular outcomes.

**What is already known on this topic**

Atopic eczema is a common systemic inflammatory condition
Previous studies have reported mixed findings for the association between atopic eczema and cardiovascular outcomes

**What this study adds**

Severe and predominantly active atopic eczema are associated with an increased risk of cardiovascular outcomes
Patients with severe atopic eczema were at a 20% increased risk of stroke, 35% to 40% increased risk of unstable angina, myocardial infarction, atrial fibrillation, and cardiovascular death, and 70% increased risk of heart failure

## Web Extra

Extra material supplied by the author

## Notes

Ethical approval: The study was approved by the Independent Scientific Advisory Committee (16_100RA) and the London School of Hygiene and Tropical Medicine (11961).

Data sharing: No additional data are available.

Transparency: The manuscripts guarantors (RJH and HJF) affirm that this manuscript is an honest, accurate, and transparent account of the study being reported; that no important aspects of the study have been omitted; and that any discrepancies from the study as planned (and, if relevant, registered) have been explained.
