# Supplementary material for: Severe and predominantly active atopic eczema in adulthood and long term risk of cardiovascular disease: population based cohort study
Source: BMJ. 2018 May 23;361:k1786. doi: 10.1136/bmj.k1786 (PMC6190010; doi:10.1136/bmj.k1786)
Supplement: Supplementary file 1 — Supplementary information: Methods S1, tables S1-S15, and figure S1 [file silr042874.ww1.pdf]

Supplementary Material

Methods S1

Further detail on defining covariates

Patients were defined as having asthma, hypertension or hyperlipidaemia on the date of their first Read code for these diagnoses. Depression was defined at first evidence of a diagnosis or symptom of depression (such as “feeling depressed” or “sad mood”); symptoms were included due to the trend post-2004 of using symptom rather than diagnosis codes in UK primary care.[1] Similarly, anxiety was defined as having a diagnosis or symptom of anxiety.

To define diabetes, we required a definite diabetes diagnosis, or a possible diabetes code (e.g. self-monitoring of blood glucose) with a subsequent diabetes-specific prescription (insulin or oral anti-diabetics), or ≥2 diabetes drug prescriptions; gestational diabetes and drug-induced diabetes were excluded. We also used age at first diagnosis, age at first treatment and treatment received to classify patients into Type 1 or 2 diabetes. Type 1 was assigned where age at first diagnosis was ≤35 years and treatment ever was exclusively insulin or where patients received at least two insulin prescriptions ≤35 years, but had no diabetes diagnosis. Type 2 was assigned where age at first diabetes diagnosis was >35 or where patients received exclusively oral anti-diabetic agents >35 years. Patients with age at diagnosis >35 but treated exclusively with insulin and those not fitting into these categories were assigned as “Unknown type”.

We selected smoking, alcohol and BMI status as follows: Data were derived from medical Read codes and data from the additional details file. Read codes classifying patients by BMI category were not used as they are very rarely recorded. Where patients had multiple recordings, the nearest status in the period -1y to +1month from cohort entry was taken (best); if not available, then the

nearest in the period +1month to +1y after cohort entry was taken (second best); if not available, then the nearest before -1y from cohort entry was taken (third best); if not available, then take nearest after +1y from cohort entry was taken (least best).

Ethnicity was assigned to five categories: White, South Asian, Black, Other or Mixed. First the most common ethnicity in CPRD was used, then the latest ethnicity in CPRD was used where several ethnicities were recorded equally, and finally HES ethnicity was used where CPRD ethnicity was missing. Since the quality of the recording for ethnicity was only acceptable from 2006 onwards, we restricted our study population to those remaining registered with the CPRD general practice after 1st January 2007[2].

Daily dose of oral corticosteroid was calculated as follow: numeric daily dose (NDD) x dose per tablet. Where NDD was missing, a “hot-deck” style imputation method was adopted, which replaced missing data with comparable data from the same set. An extra binary variable for quantity of tablets per prescription was created, categorising quantity about the median number (42) into low and high. If a patient had any other record with the same quantity and dose per tablet, the median NDD among those records was used where NDD was missing. If a patient had no recorded NDD but had any other record of the same dose per tablet and quantity as a binary variable, the median NDD among those records was used. If a patient did not have a recorded NDD or quantity, but had records for the same dose per tablet, then the median NDD among those records was used. If there was no record of NDD, dose per tablet or quantity, but there were other patients in the dataset in the same 5-year age band, of the same gender, with the same dose per tablet and quantity, the median NDD for those records was used. Finally, if none of the above were possible, patients in the dataset in the same 5-year age band, of the same gender, with the same dose per tablet and quantity as a binary variable, the median NDD among these records was used.

References

1. Rait, G., et al., *Recent trends in the incidence of recorded depression in primary care.* Br J Psychiatry, 2009. **195**(6): p. 520-4.
2. Mathur, R., et al., *Completeness and usability of ethnicity data in UK-based primary care and hospital databases.* J Public Health (Oxf), 2014. **36**(4): p. 684-92.

Table S1. Summary statistics for covariates. Mean (%) unless otherwise stated.

	Overall sample			Complete data on analysis variables			From valid matched set[1]		
	Without atopic eczema n = 2,169,123 (82.3%)	With atopic eczema n = 466,883 (17.7%)	Total n = 2,636,006	Without atopic eczema n = 1,726,339 (81.5%)	With atopic eczema n = 392,223 (18.5%)	Total n = 2,118,562	Without atopic eczema n = 1,528,477 (79.8%)	With atopic eczema n = 387,439 (20.2%)	Total n = 1,915,916
Follow-up (years)									
Median	4.34	5.10	4.49	4.81	5.67	4.95	4.94	5.69	5.08
Quartiles	1.69, 8.75	2.07, 9.72	1.75, 8.91	1.90, 9.41	2.39, 10.31	1.98, 9.59	1.97, 9.57	2.40, 10.34	2.05, 9.75
**At entry to cohort**									
Gender									
Male	868,162 (40.0)	187,231 (40.1)	1,055,393 (40.0)	612,425 (35.5)	142,772 (36.4)	755,197 (35.6)	511,676 (33.5)	139,908 (36.1)	651,584 (34.0)
Female	1,300,961 (60.0)	279,652 (59.9)	1,580,613 (60.0)	1,113,914 (64.5)	249,451 (63.6)	1,363,365 (64.4)	1,016,801 (66.5)	247,531 (63.9)	1,264,332 (66.0)
Age (years)									
18-19	305,114 (14.1)	77,685 (16.6)	382,799 (14.5)	138,107 (8.0)	38,824 (9.9)	176,931 (8.4)	87,600 (5.7)	36,392 (9.4)	123,992 (6.5)
20-29	439,343 (20.3)	80,625 (17.3)	519,968 (19.7)	338,586 (19.6)	66,806 (17.0)	405,392 (19.1)	275,632 (18.0)	66,156 (17.1)	341,788 (17.8)
30-39	407,927 (18.8)	76,850 (16.5)	484,777 (18.4)	348,326 (20.2)	70,389 (17.9)	418,715 (19.8)	315,206 (20.6)	70,222 (18.1)	385,428 (20.1)
40-49	327,908 (15.1)	66,212 (14.2)	394,120 (15.0)	289,313 (16.8)	61,977 (15.8)	351,290 (16.6)	272,436 (17.8)	61,898 (16.0)	334,334 (17.5)
50-59	282,612 (13.0)	58,223 (12.5)	340,835 (12.9)	255,015 (14.8)	55,398 (14.1)	310,413 (14.7)	243,469 (15.9)	55,294 (14.3)	298,763 (15.6)
60-69	223,110 (10.3)	51,812 (11.1)	274,922 (10.4)	204,588 (11.9)	49,569 (12.6)	254,157 (12.0)	195,936 (12.8)	49,420 (12.8)	245,356 (12.8)
70-79	127,765 (5.9)	36,918 (7.9)	164,683 (6.2)	112,296 (6.5)	34,232 (8.7)	146,528 (6.9)	104,132 (6.8)	33,837 (8.7)	137,969 (7.2)
80+	55,344 (2.6)	18,558 (4.0)	73,902 (2.8)	40,108 (2.3)	15,028 (3.8)	55,136 (2.6)	34,066 (2.2)	14,220 (3.7)	48,286 (2.5)
IMD									
1 (Least deprived)	519,806 (24.0)	113,826 (24.4)	633,632 (24.1)	416,116 (24.1)	95,441 (24.3)	511,557 (24.1)	370,953 (24.3)	94,223 (24.3)	465,176 (24.3)
2	469,320 (21.7)	101,484 (21.8)	570,804 (21.7)	375,026 (21.7)	85,349 (21.8)	460,375 (21.7)	332,853 (21.8)	84,297 (21.8)	417,150 (21.8)
3	446,757 (20.6)	95,075 (20.4)	541,832 (20.6)	355,055 (20.6)	79,622 (20.3)	434,677 (20.5)	312,836 (20.5)	78,603 (20.3)	391,439 (20.4)
4	386,924 (17.9)	82,716 (17.7)	469,640 (17.8)	309,280 (17.9)	70,030 (17.9)	379,310 (17.9)	273,332 (17.9)	69,223 (17.9)	342,555 (17.9)
5 (Most deprived)	343,236 (15.8)	73,198 (15.7)	416,434 (15.8)	270,862 (15.7)	61,781 (15.8)	332,643 (15.7)	238,503 (15.6)	61,093 (15.8)	299,596 (15.6)
BMI									
Underweight	56,701 (3.3)	12,085 (3.1)	68,786 (3.2)	55,988 (3.2)	11,971 (3.1)	67,959 (3.2)	45,488 (3.0)	11,590 (3.0)	57,078 (3.0)
Normal weight	818,457 (47.2)	177,338 (45.1)	995,795 (46.8)	813,510 (47.1)	176,677 (45.0)	990,187 (46.7)	709,903 (46.4)	174,234 (45.0)	884,137 (46.1)
Overweight	540,618 (31.1)	124,906 (31.7)	665,524 (31.3)	538,207 (31.2)	124,547 (31.8)	662,754 (31.3)	484,434 (31.7)	123,352 (31.8)	607,786 (31.7)
Obese	320,009 (18.4)	79,276 (20.1)	399,285 (18.8)	318,634 (18.5)	79,028 (20.1)	397,662 (18.8)	288,652 (18.9)	78,263 (20.2)	366,915 (19.2)
Smoking									
Non-smoker	979,824 (48.0)	208,478 (45.8)	1,188,302 (47.6)	799,258 (46.3)	170,823 (43.6)	970,081 (45.8)	699,570 (45.8)	168,221 (43.4)	867,791 (45.3)
Current smoker	644,991 (31.6)	135,282 (29.7)	780,273 (31.2)	545,655 (31.6)	117,757 (30.0)	663,412 (31.3)	480,780 (31.5)	116,551 (30.1)	597,331 (31.2)
Ex-smoker	417,349 (20.4)	111,402 (24.5)	528,751 (21.2)	381,426 (22.1)	103,643 (26.4)	485,069 (22.9)	348,127 (22.8)	102,667 (26.5)	450,794 (23.5)
Diabetes	57,614 (2.7)	16,333 (3.5)	73,947 (2.8)	55,612 (3.2)	15,974 (4.1)	71,586 (3.4)	51,213 (3.4)	15,777 (4.1)	66,990 (3.5)
Hypertension	214,181 (9.9)	61,413 (13.2)	275,594 (10.5)	202,558 (11.7)	58,654 (15.0)	261,212 (12.3)	190,217 (12.4)	58,001 (15.0)	248,218 (13.0)

Hyperlipidaemia	64,720 (3.0)	19,976 (4.3)	84,696 (3.2)	62,571 (3.6)	19,524 (5.0)	82,095 (3.9)	59,376 (3.9)	19,342 (5.0)	78,718 (4.1)
Depression	367,805 (17.0)	104,014 (22.3)	471,819 (17.9)	329,132 (19.1)	95,844 (24.4)	424,976 (20.1)	300,699 (19.7)	95,131 (24.6)	395,830 (20.7)
Anxiety	238,393 (11.0)	71,754 (15.4)	310,147 (11.8)	212,853 (12.3)	65,786 (16.8)	278,639 (13.2)	194,289 (12.7)	65,248 (16.8)	259,537 (13.5)
Asthma	273,492 (12.6)	113,685 (24.3)	387,177 (14.7)	222,844 (12.9)	93,751 (23.9)	316,595 (14.9)	190,728 (12.5)	91,955 (23.7)	282,683 (14.8)
Severe alcohol use	36,327 (1.7)	9,588 (2.1)	45,915 (1.7)	32,097 (1.9)	8,792 (2.2)	40,889 (1.9)	28,803 (1.9)	8,730 (2.3)	37,533 (2.0)
**By exit from cohort**									
Diabetes	118,067 (5.4)	33,489 (7.2)	151,556 (5.7)	114,840 (6.7)	32,866 (8.4)	147,706 (7.0)	106,106 (6.9)	32,574 (8.4)	138,680 (7.2)
Hypertension	336,134 (15.5)	92,062 (19.7)	428,196 (16.2)	319,482 (18.5)	88,374 (22.5)	407,856 (19.3)	299,283 (19.6)	87,564 (22.6)	386,847 (20.2)
Hyperlipidaemia	134,610 (6.2)	38,758 (8.3)	173,368 (6.6)	130,552 (7.6)	37,916 (9.7)	168,468 (8.0)	123,187 (8.1)	37,652 (9.7)	160,839 (8.4)
Depression	531,394 (24.5)	147,338 (31.6)	678,732 (25.7)	472,628 (27.4)	134,549 (34.3)	607,177 (28.7)	426,864 (27.9)	133,394 (34.4)	560,258 (29.2)
Anxiety	372,595 (17.2)	108,543 (23.2)	481,138 (18.3)	332,918 (19.3)	99,270 (25.3)	432,188 (20.4)	301,356 (19.7)	98,410 (25.4)	399,766 (20.9)
Asthma	318,867 (14.7)	128,438 (27.5)	447,305 (17.0)	265,527 (15.4)	107,716 (27.5)	373,243 (17.6)	229,455 (15.0)	105,810 (27.3)	335,265 (17.5)
Severe alcohol use	60,203 (2.8)	15,798 (3.4)	76,001 (2.9)	53,609 (3.1)	14,448 (3.7)	68,057 (3.2)	48,121 (3.1)	14,337 (3.7)	62,458 (3.3)

[1]Matched sets including one exposed patient and at least one unexposed patient.

Table S2. Association between atopic eczema and cardiovascular outcomes, by gender. Fitted to patients with complete data for all variables included in the models and from valid matched sets[1]. n = 1,915,916 (1,842,759 unique patients).

	n	P-Y at risk	Events	Adjusted HR and 99% CI[2]		Interaction p-value
**Primary outcomes**						
Myocardial infarction						0.08
Males						
Unexposed	511,676	3,176,993	9,976	1.00	(ref)	
Exposed	139,908	917,775	3,191	1.04	0.93, 1.15	
Females						
Unexposed	1,016,801	6,184,529	7,202	1.00	(ref)	
Exposed	247,531	1,651,439	2,370	1.10	0.96, 1.26	
Unstable angina						0.25
Males						
Unexposed	511,676	3,199,765	3,533	1.00	(ref)	
Exposed	139,908	924,356	1,238	1.27	1.08, 1.49	
Females						
Unexposed	1,016,801	6,192,605	3,526	1.00	(ref)	
Exposed	247,531	1,653,808	1,222	1.21	1.01, 1.45	
Heart failure						0.71
Males						
Unexposed	511,676	3,193,535	7,704	1.00	(ref)	
Exposed	139,908	921,122	3,101	1.22	1.08, 1.37	
Females						
Unexposed	1,016,801	6,181,849	9,279	1.00	(ref)	
Exposed	247,531	1,649,290	3,340	1.17	1.04, 1.31	
Atrial fibrillation						0.83
Males						
Unexposed	511,676	3,164,721	13,364	1.00	(ref)	
Exposed	139,908	912,022	4,860	1.12	1.02, 1.23	
Females						
Unexposed	1,016,801	6,151,610	15,207	1.00	(ref)	
Exposed	247,531	1,640,289	5,032	1.09	1.00, 1.20	
Stroke						0.30
Males						
Unexposed	511,676	3,187,269	9,202	1.00	(ref)	
Exposed	139,908	920,739	3,169	1.14	1.02, 1.27	
Females						
Unexposed	1,016,801	6,173,983	12,185	1.00	(ref)	
Exposed	247,531	1,648,010	3,980	1.07	0.97, 1.19	
Cardiovascular death						0.23
Males						
Unexposed	511,676	3,217,261	13,843	1.00	(ref)	
Exposed	139,908	930,605	5,309	1.04	0.94, 1.14	

Females						
Unexposed	1,016,801	6,210,159	16,273	1.00	(ref)	
Exposed	247,531	1,659,700	5,504	0.93	0.85, 1.03	
**Secondary outcome**						
Coronary revascularisation						0.47
Males						
Unexposed	511,676	3,171,599	10,540	1.00	(ref)	
Exposed	139,908	915,741	3,278	1.11	1.00, 1.23	
Females						
Unexposed	1,016,801	6,186,782	5,655	1.00	(ref)	
Exposed	247,531	1,652,191	1,778	1.20	1.03, 1.39	

[1]Matched sets including one exposed patient and at least one unexposed patient.

[2]Estimated hazard ratios from Cox regression with current age as underlying timescale, stratified by matched set (matched on age at cohort entry, gender, date at cohort entry and practice).

Adjusted: Adjusted for current calendar period (1997-1999, 2000-2004, 2005-2009, 2010-2015), time since diagnosis (0-4, 5-9, 10-14, 15-19, 20+ years), IMD at cohort entry, and time-varying asthma.

Table S3. Association between atopic eczema and cardiovascular outcomes, by exposure to time-varying asthma. Fitted to patients with complete data for all variables included in the models and from valid matched sets[1]. n = 1,915,916 (1,842,759 unique patients).

	n	P-Y at risk	Events	Adjusted HR and 99% CI[2]		Interaction p-value
**Primary outcomes**						
Myocardial infarction						0.71
No asthma						
Unexposed	1,337,737	8,082,456	14,692	1.00	(ref)	
Exposed	295,403	1,921,190	4,326	1.07	0.97, 1.17	
Asthma						
Unexposed	229,121	1,279,066	2,486	1.00	(ref)	
Exposed	105,688	648,024	1,235	1.06	0.72, 1.54	
Unstable angina						0.57
No asthma						
Unexposed	1,337,737	8,109,748	5,881	1.00	(ref)	
Exposed	295,403	1,928,551	1,866	1.32	1.15, 1.52	
Asthma						
Unexposed	229,227	1,282,622	1,178	1.00	(ref)	
Exposed	105,710	649,614	594	1.04	0.62, 1.75	
Heart failure						0.26
No asthma						
Unexposed	1,337,737	8,097,247	13,820	1.00	(ref)	
Exposed	295,403	1,923,192	4,829	1.22	1.11, 1.34	
Asthma						
Unexposed	229,043	1,278,137	3,163	1.00	(ref)	
Exposed	105,634	647,220	1,612	1.32	0.94, 1.86	
Atrial fibrillation						0.42
No asthma						
Unexposed	1,337,737	8,045,565	24,074	1.00	(ref)	
Exposed	295,403	1,908,488	7,670	1.12	1.03, 1.20	
Asthma						
Unexposed	228,919	1,270,766	4,497	1.00	(ref)	
Exposed	105,578	643,823	2,222	1.15	0.86, 1.55	
Stroke						0.52
No asthma						
Unexposed	1,337,737	8,081,588	18,521	1.00	(ref)	
Exposed	295,403	1,920,407	5,767	1.13	1.04, 1.23	
Asthma						
Unexposed	229,166	1,279,664	2,866	1.00	(ref)	
Exposed	105,706	648,342	1,382	0.99	0.68, 1.43	
Cardiovascular death						0.21
No asthma						
Unexposed	1,337,737	8,138,548	25,215	1.00	(ref)	
Exposed	295,403	1,937,496	8,372	1.00	0.92, 1.08	

Asthma						
Unexposed	229,442	1,288,871	4,901	1.00	(ref)	
Exposed	105,801	652,809	2,441	0.98	0.72, 1.34	
**Secondary outcome**						
Coronary revascularisation						0.33
No asthma						
Unexposed	1,337,737	8,079,056	14,012	1.00	(ref)	
Exposed	295,403	1,919,729	4,004	1.17	1.06, 1.28	
Asthma						
Unexposed	229,117	1,279,325	2,183	1.00	(ref)	
Exposed	105,694	648,203	1,052	0.87	0.57, 1.33	

[1]Matched sets including one exposed patient and at least one unexposed patient.

[2]Estimated hazard ratios from Cox regression with current age as underlying timescale, stratified by matched set (matched on age at cohort entry, gender, date at cohort entry and practice).

Adjusted: Adjusted for current calendar period (1997-1999, 2000-2004, 2005-2009, 2010-2015), time since diagnosis (0-4, 5-9, 10-14, 15-19, 20+ years), IMD at cohort entry, and time-varying asthma.

Table S4. Association between atopic eczema and cardiovascular outcomes, by current age. Fitted to patients with complete data for all variables included in the models and from valid matched sets[1] n = 1,915,916 (1,842,759 unique patients).

	n	P-Y at risk	Events	Adjusted HR and 99% CI[2]		Interaction p-value
**Primary outcomes**						
Myocardial infarction						0.50
Age 18-39 years						
Unexposed	674,921	2,696,041	193	1.00	(ref)	
Exposed	172,502	791,444	52	0.81	0.35, 1.83	
Age 40-59 years						
Unexposed	695,301	3,644,129	3,852	1.00	(ref)	
Exposed	163,309	911,424	1,051	1.07	0.90, 1.27	
Age 60+ years						
Unexposed	506,685	3,021,353	13,133	1.00	(ref)	
Exposed	139,125	866,346	4,458	1.07	0.97, 1.17	
Unstable angina						0.51
Age 18-39 years						
Unexposed	674,921	2,696,223	109	1.00	(ref)	
Exposed	172,502	791,468	38	1.52	0.60, 3.89	
Age 40-59 years						
Unexposed	695,333	3,649,980	1,874	1.00	(ref)	
Exposed	163,312	912,682	579	1.35	1.07, 1.69	
Age 60+ years						
Unexposed	507,315	3,046,167	5,076	1.00	(ref)	
Exposed	139,254	874,015	1,843	1.20	1.04, 1.38	
Heart failure						0.14
Age 18-39 years						
Unexposed	674,921	2,696,062	154	1.00	(ref)	
Exposed	172,502	791,383	64	0.99	0.40, 2.43	
Age 40-59 years						
Unexposed	695,360	3,653,137	1,551	1.00	(ref)	
Exposed	163,317	913,437	514	1.53	1.19, 1.97	
Age 60+ years						
Unexposed	507,487	3,026,184	15,278	1.00	(ref)	
Exposed	139,312	865,591	5,863	1.16	1.06, 1.26	
Atrial fibrillation						0.74
Age 18-39 years						
Unexposed	674,921	2,695,393	354	1.00	(ref)	
Exposed	172,502	791,144	129	1.30	0.76, 2.23	
Age 40-59 years						
Unexposed	695,241	3,647,045	3,080	1.00	(ref)	
Exposed	163,280	912,122	878	1.19	0.98, 1.44	
Age 60+ years						
Unexposed	506,706	2,973,893	25,137	1.00	(ref)	
Exposed	139,114	849,045	8,885	1.09	1.02, 1.17	

Stroke						0.35
Age 18-39 years						
Unexposed	674,921	2,695,012	521	1.00	(ref)	
Exposed	172,502	791,099	152	1.32	0.86, 2.05	
Age 40-59 years						
Unexposed	695,232	3,647,667	3,170	1.00	(ref)	
Exposed	163,278	912,205	876	1.15	0.94, 1.40	
Age 60+ years						
Unexposed	507,035	3,018,573	17,696	1.00	(ref)	
Exposed	139,235	865,445	6,121	1.08	1.00, 1.18	
Cardiovascular death						0.003
Age 18-39 years						
Unexposed	674,921	2,696,477	239	1.00	(ref)	
Exposed	172,502	791,554	79	1.03	0.53, 2.01	
Age 40-59 years						
Unexposed	695,408	3,657,531	2,269	1.00	(ref)	
Exposed	163,335	915,088	715	1.27	1.02, 1.57	
Age 60+ years						
Unexposed	508,016	3,073,412	27,608	1.00	(ref)	
Exposed	139,483	883,662	10,019	0.95	0.88, 1.03	
**Secondary outcome**						
Coronary revascularisation						0.63
Age 18-39 years						
Unexposed	674,921	2,695,764	307	1.00	(ref)	
Exposed	172,502	791,296	108	1.50	0.88, 2.57	
Age 40-59 years						
Unexposed	695,280	3,643,598	4,438	1.00	(ref)	
Exposed	163,299	910,984	1,210	1.15	0.98, 1.35	
Age 60+ years						
Unexposed	506,437	3,019,020	11,450	1.00	(ref)	
Exposed	139,058	865,652	3,738	1.12	1.01, 1.24	

[1]Matched sets including one exposed patient and at least one unexposed patient.
[2]Estimated hazard ratios from Cox regression with current age as underlying timescale, stratified by matched set (matched on age at cohort entry, gender, date at cohort entry and practice).
Adjusted: Adjusted for current calendar period (1997-1999, 2000-2004, 2005-2009, 2010-2015), time since diagnosis (0-4, 5-9, 10-14, 15-19, 20+ years), IMD at cohort entry, and time-varying asthma.

Table S5. Association between atopic eczema and cardiovascular outcomes, by severity of atopic eczema. Fitted to patients with complete data for all variables included in the models and from valid matched sets[1]. n = 1,915,916 (1,842,759 unique patients).

				Hazard ratio and 99% confidence interval[2]					
	n	P-Y at risk	Events	Unadjusted		Adjusted		Mediation model	
**Primary outcomes**									
Myocardial infarction									
Unexposed	1,528,477	9,361,522	17,178	1.00	(ref)	1.00	(ref)	1.00	(ref)
Exposed – mild	285,661	1,544,463	2,751	1.03	0.96, 1.09	1.00	0.91, 1.10	1.00	0.91, 1.10
Exposed – moderate	145,648	900,472	2,447	1.15	1.07, 1.23	1.11	1.01, 1.23	1.07	0.97, 1.18
Exposed – severe	19,635	124,279	363	1.46	1.21, 1.74	1.41	1.15, 1.71	1.37	1.12, 1.68
Unstable angina									
Unexposed	1,528,477	9,392,370	7,059	1.00	(ref)	1.00	(ref)	1.00	(ref)
Exposed – mild	285,661	1,548,178	1,319	1.22	1.11, 1.33	1.25	1.10, 1.43	1.19	1.04, 1.37
Exposed – moderate	145,748	904,975	1,004	1.20	1.08, 1.34	1.22	1.06, 1.42	1.11	0.96, 1.29
Exposed – severe	19,661	125,012	137	1.47	1.10, 1.97	1.48	1.08, 2.03	1.41	1.02, 1.95
Heart failure									
Unexposed	1,528,477	9,375,383	16,983	1.00	(ref)	1.00	(ref)	1.00	(ref)
Exposed – mild	285,661	1,545,238	3,081	1.12	1.05, 1.19	1.12	1.02, 1.23	1.12	1.02, 1.24
Exposed – moderate	145,614	900,749	2,941	1.27	1.19, 1.35	1.25	1.14, 1.38	1.20	1.09, 1.33
Exposed – severe	19,624	124,425	419	1.71	1.43, 2.05	1.69	1.38, 2.06	1.67	1.36, 2.05
Atrial fibrillation									
Unexposed	1,528,477	9,316,331	28,571	1.00	(ref)	1.00	(ref)	1.00	(ref)
Exposed – mild	285,661	1,536,766	4,922	1.05	1.00, 1.10	1.05	0.97, 1.13	1.02	0.95, 1.10
Exposed – moderate	145,377	892,059	4,410	1.18	1.12, 1.24	1.17	1.08, 1.27	1.12	1.04, 1.21
Exposed – severe	19,575	123,487	560	1.40	1.21, 1.62	1.38	1.17, 1.62	1.35	1.14, 1.59
Stroke									
Unexposed	1,528,477	9,361,252	21,387	1.00	(ref)	1.00	(ref)	1.00	(ref)
Exposed – mild	285,661	1,543,768	3,598	1.03	0.98, 1.09	1.07	0.98, 1.16	1.06	0.97, 1.15

Exposed – moderate	145,627	900,587	3,162	1.11	1.04, 1.18	1.14	1.04, 1.25	1.09	1.00, 1.20
Exposed – severe	19,622	124,394	389	1.19	1.00, 1.42	1.22	1.01, 1.48	1.20	0.99, 1.46
Cardiovascular death									
Unexposed	1,528,477	9,427,420	30,116	1.00	(ref)	1.00	(ref)	1.00	(ref)
Exposed – mild	285,661	1,554,072	5,005	0.97	0.92, 1.02	0.90	0.84, 0.98	0.90	0.83, 0.98
Exposed – moderate	145,947	910,385	5,156	1.15	1.09, 1.21	1.06	0.98, 1.15	1.01	0.93, 1.10
Exposed – severe	19,696	125,849	652	1.49	1.29, 1.73	1.38	1.17, 1.62	1.30	1.10, 1.53
**Secondary outcome**									
Coronary revascularisation									
Unexposed	1,528,477	9,358,381	16,195	1.00	(ref)	1.00	(ref)	1.00	(ref)
Exposed – mild	285,661	1,543,507	2,597	1.09	1.02, 1.16	1.12	1.02, 1.23	1.08	0.98, 1.19
Exposed – moderate	145,609	900,137	2,141	1.11	1.04, 1.20	1.14	1.03, 1.26	1.05	0.95, 1.17
Exposed – severe	19,624	124,288	318	1.42	1.17, 1.71	1.47	1.19, 1.81	1.36	1.10, 1.69

[1]Matched sets including one exposed patient and at least one unexposed patient.

[2]Estimated hazard ratios from Cox regression with current age as underlying timescale, stratified by matched set (matched on age at cohort entry, gender, date at cohort entry and practice).

Unadjusted: No adjustment.

Adjusted: Adjusted for current calendar period (1997-1999, 2000-2004, 2005-2009, 2010-2015), time since diagnosis (0-4, 5-9, 10-14, 15-19, 20+ years), IMD at cohort entry, and time-varying asthma.

Mediation model: Adjusted additionally for BMI and smoking at cohort entry, and time-varying hyperlipidaemia, hypertension, depression, anxiety, diabetes and severe alcohol use.

Table S6. Association between atopic eczema and cardiovascular outcomes, by activity of atopic eczema. Fitted to patients with complete data for all variables included in the models and from valid matched sets[1]. n = 1,915,916 (1,842,759 unique patients).

	n	P-Y at risk	Events	Hazard ratio and 99% confidence interval[2]					
				Unadjusted		Adjusted		Mediation model	
**Primary outcomes**									
Myocardial infarction									
Unexposed	1,528,477	9,361,522	17,178	1.00	(ref)	1.00	(ref)	1.00	(ref)
Exposed – never active	122,305	672,569	774	0.96	0.86, 1.08	0.92	0.80, 1.06	0.95	0.83, 1.10
Exposed – <50% active	137,510	1,212,382	2,340	1.00	0.93, 1.07	0.95	0.86, 1.05	0.94	0.85, 1.04
Exposed – 50+% active	127,624	684,263	2,447	1.29	1.20, 1.38	1.20	1.09, 1.32	1.16	1.05, 1.27
Unstable angina									
Unexposed	1,528,477	9,392,370	7,059	1.00	(ref)	1.00	(ref)	1.00	(ref)
Exposed – never active	122,305	673,925	301	0.86	0.72, 1.03	0.89	0.72, 1.10	0.88	0.70, 1.10
Exposed – <50% active	137,510	1,216,812	1,047	1.11	1.01, 1.23	1.12	0.97, 1.29	1.05	0.91, 1.22
Exposed – 50+% active	127,624	687,428	1,112	1.56	1.40, 1.73	1.49	1.30, 1.72	1.36	1.18, 1.58
Heart failure									
Unexposed	1,528,477	9,375,383	16,983	1.00	(ref)	1.00	(ref)	1.00	(ref)
Exposed – never active	122,305	673,431	653	0.89	0.78, 1.01	0.88	0.76, 1.03	0.92	0.79, 1.08
Exposed – <50% active	137,510	1,214,652	2,352	0.99	0.93, 1.07	0.94	0.85, 1.05	0.96	0.86, 1.07
Exposed – 50+% active	127,624	682,329	3,436	1.55	1.46, 1.65	1.43	1.30, 1.56	1.37	1.24, 1.50
Atrial fibrillation									
Unexposed	1,528,477	9,316,331	28,571	1.00	(ref)	1.00	(ref)	1.00	(ref)
Exposed – never active	122,305	671,221	1,165	0.94	0.86, 1.04	0.93	0.83, 1.05	0.93	0.83, 1.05
Exposed – <50% active	137,510	1,204,930	4,230	1.04	0.99, 1.10	1.02	0.94, 1.10	1.00	0.92, 1.08
Exposed – 50+% active	127,624	676,161	4,497	1.27	1.21, 1.34	1.22	1.13, 1.31	1.17	1.08, 1.26
Stroke									
Unexposed	1,528,477	9,361,252	21,387	1.00	(ref)	1.00	(ref)	1.00	(ref)
Exposed – never active	122,305	672,590	1,039	1.07	0.96, 1.18	1.10	0.97, 1.24	1.12	0.99, 1.27

Exposed – <50% active	137,510	1,212,702	2,819	0.93	0.87, 0.99	0.94	0.86, 1.03	0.93	0.85, 1.02
Exposed – 50+% active	127,624	683,457	3,291	1.24	1.17, 1.32	1.24	1.14, 1.35	1.19	1.09, 1.30
Cardiovascular death									
Unexposed	1,528,477	9,427,420	30,116	1.00	(ref)	1.00	(ref)	1.00	(ref)
Exposed – never active	122,305	675,158	1,637	1.36	1.24, 1.48	1.23	1.10, 1.38	1.27	1.13, 1.43
Exposed – <50% active	137,510	1,222,542	3,476	0.75	0.71, 0.80	0.67	0.61, 0.73	0.66	0.60, 0.72
Exposed – 50+% active	127,624	692,605	5,700	1.32	1.26, 1.39	1.16	1.07, 1.25	1.10	1.02, 1.19
**Secondary outcome**									
Coronary revascularisation									
Unexposed	1,528,477	9,358,381	16,195	1.00	(ref)	1.00	(ref)	1.00	(ref)
Exposed – never active	122,305	672,352	710	0.90	0.80, 1.01	0.90	0.79, 1.04	0.91	0.79, 1.05
Exposed – <50% active	137,510	1,211,346	2,354	1.09	1.02, 1.16	1.09	0.99, 1.21	1.05	0.94, 1.16
Exposed – 50+% active	127,624	684,234	1,992	1.27	1.18, 1.37	1.26	1.14, 1.40	1.16	1.05, 1.29

[1]Matched sets including one exposed patient and at least one unexposed patient.

[2]Estimated hazard ratios from Cox regression with current age as underlying timescale, stratified by matched set (matched on age at cohort entry, gender, date at cohort entry and practice).

Unadjusted: No adjustment.

Adjusted: Adjusted for current calendar period (1997-1999, 2000-2004, 2005-2009, 2010-2015), time since diagnosis (0-4, 5-9, 10-14, 15-19, 20+ years), IMD at cohort entry, and time-varying asthma.

Mediation model: Adjusted additionally for BMI and smoking at cohort entry, and time-varying hyperlipidaemia, hypertension, depression, anxiety, diabetes and severe alcohol use.

Table S7. Association between atopic eczema and cardiovascular outcomes, by activity of atopic eczema. Restricted to patients with at least 5 years of follow-up. Fitted to patients with complete data for all variables included in the models, from valid matched sets[1].

	n	Unique patients	n	P-Y at risk	Events	Hazard ratio and 99% confidence interval[2]					
						Unadjusted		Adjusted		Mediation model	
**Primary outcomes**											
Myocardial infarction	804,018	796,263									
Unexposed			603,987	6,213,289	6,840	1.00	(ref)	1.00	(ref)	1.00	(ref)
Exposed – never active			49,727	497,892	352	0.96	0.81, 1.14	0.90	0.74, 1.10	0.94	0.76, 1.15
Exposed – <50% active			99,145	1,062,387	1,243	0.95	0.87, 1.04	0.90	0.78, 1.02	0.88	0.77, 1.01
Exposed – 50+% active			51,159	512,074	1,039	1.26	1.13, 1.40	1.15	1.00, 1.33	1.10	0.95, 1.27
Unstable angina	808,619	800,778									
Unexposed			607,788	6,261,278	2,543	1.00	(ref)	1.00	(ref)	1.00	(ref)
Exposed – never active			49,850	499,596	126	0.85	0.64, 1.12	0.86	0.62, 1.20	0.88	0.62, 1.24
Exposed – <50% active			99,516	1,067,742	510	1.15	0.99, 1.33	1.15	0.94, 1.41	1.10	0.89, 1.36
Exposed – 50+% active			51,465	516,016	452	1.55	1.31, 1.83	1.48	1.19, 1.83	1.39	1.11, 1.74
Heart failure	805,596	797,803									
Unexposed			605,495	6,234,414	6,860	1.00	(ref)	1.00	(ref)	1.00	(ref)
Exposed – never active			49,793	499,076	257	0.74	0.61, 0.91	0.79	0.63, 1.00	0.83	0.65, 1.05
Exposed – <50% active			99,334	1,064,930	1,403	1.08	0.98, 1.18	1.10	0.96, 1.25	1.11	0.97, 1.26
Exposed – 50+% active			50,974	510,092	1,490	1.53	1.39, 1.69	1.50	1.31, 1.70	1.42	1.24, 1.63
Atrial fibrillation	797,614	789,960									
Unexposed			598,885	6,150,085	12,339	1.00	(ref)	1.00	(ref)	1.00	(ref)
Exposed – never active			49,616	496,375	578	0.95	0.82, 1.09	0.94	0.80, 1.11	0.96	0.82, 1.13
Exposed – <50% active			98,643	1,053,441	2,502	1.06	0.99, 1.14	1.04	0.95, 1.15	1.03	0.93, 1.14
Exposed – 50+% active			50,470	502,347	2,099	1.24	1.14, 1.34	1.19	1.08, 1.32	1.15	1.04, 1.28
Stroke	804,146	796,375									
Unexposed			604,142	6,216,350	8,927	1.00	(ref)	1.00	(ref)	1.00	(ref)
Exposed – never active			49,736	498,081	437	0.99	0.84, 1.15	1.02	0.85, 1.22	1.06	0.88, 1.28

Exposed – <50% active			99,186	1,062,765	1,652	0.97	0.89, 1.05	0.99	0.88, 1.12	0.99	0.87, 1.11
Exposed – 50+% active			51,082	511,143	1,450	1.17	1.07, 1.28	1.19	1.05, 1.34	1.14	1.01, 1.29
Cardiovascular death	814,798	806,886									
Unexposed			612,910	6,324,719	13,779	1.00	(ref)	1.00	(ref)	1.00	(ref)
Exposed – never active			49,965	501,131	661	1.07	0.93, 1.22	1.07	0.92, 1.25	1.14	0.97, 1.34
Exposed – <50% active			99,990	1,074,795	2,506	0.90	0.84, 0.97	0.88	0.80, 0.97	0.87	0.79, 0.96
Exposed – 50+% active			51,933	522,202	2,834	1.30	1.21, 1.40	1.23	1.12, 1.35	1.18	1.07, 1.30
**Secondary outcome**											
Coronary revascularisation	803,991	796,231									
Unexposed			603,887	6,212,162	7,188	1.00	(ref)	1.00	(ref)	1.00	(ref)
Exposed – never active			49,738	497,800	368	0.91	0.77, 1.07	0.90	0.74, 1.09	0.92	0.75, 1.12
Exposed – <50% active			99,164	1,061,419	1,365	1.05	0.96, 1.15	1.05	0.92, 1.19	1.01	0.89, 1.15
Exposed – 50+% active			51,202	512,119	916	1.22	1.09, 1.36	1.21	1.05, 1.39	1.13	0.98, 1.30

[1]Matched sets including one exposed patient and at least one unexposed patient.

[2]Estimated hazard ratios from Cox regression with current age as underlying timescale, stratified by matched set (matched on age at cohort entry, gender, date at cohort entry and practice).

Unadjusted: No adjustment.

Adjusted: Adjusted for current calendar period (1997-1999, 2000-2004, 2005-2009, 2010-2015), time since diagnosis (0-4, 5-9, 10-14, 15-19, 20+ years), IMD at cohort entry, and time-varying asthma.

Mediation model: Adjusted additionally for BMI and smoking at cohort entry, and time-varying hyperlipidaemia, hypertension, depression, anxiety, diabetes and severe alcohol use.

Table S8. Association between atopic eczema and cardiovascular outcomes, with exposed patients restricted to those with incident atopic eczema. Fitted to patients with complete data for all variables included in the models and from valid matched sets[1]. n = 1,042,765 (1,005,892 unique patients).

				Hazard ratio and 99% confidence interval[2]					
	n	P-Y at risk	Events	Unadjusted		Adjusted		Mediation model	
**Primary outcomes**									
Myocardial infarction									
Unexposed	834,643	4,607,565	8,809	1.00	(ref)	1.00	(ref)	1.00	(ref)
Exposed	208,122	1,253,178	2,829	1.08	1.02, 1.15	1.06	0.97, 1.16	1.04	0.95, 1.14
Unstable angina									
Unexposed	834,643	4,623,203	3,401	1.00	(ref)	1.00	(ref)	1.00	(ref)
Exposed	208,122	1,257,505	1,198	1.25	1.13, 1.38	1.28	1.12, 1.47	1.20	1.04, 1.38
Heart failure									
Unexposed	834,643	4,615,657	8,125	1.00	(ref)	1.00	(ref)	1.00	(ref)
Exposed	208,122	1,254,121	3,165	1.19	1.12, 1.27	1.18	1.07, 1.29	1.16	1.05, 1.28
Atrial fibrillation									
Unexposed	834,643	4,585,819	14,918	1.00	(ref)	1.00	(ref)	1.00	(ref)
Exposed	208,122	1,244,931	5,324	1.11	1.06, 1.17	1.10	1.02, 1.18	1.06	0.99, 1.14
Stroke									
Unexposed	834,643	4,607,604	10,877	1.00	(ref)	1.00	(ref)	1.00	(ref)
Exposed	208,122	1,252,777	3,688	1.06	1.00, 1.12	1.10	1.01, 1.19	1.07	0.98, 1.17
Cardiovascular death									
Unexposed	834,643	4,638,169	14,784	1.00	(ref)	1.00	(ref)	1.00	(ref)
Exposed	208,122	1,262,895	5,273	1.00	0.95, 1.05	0.96	0.89, 1.04	0.94	0.86, 1.01
**Secondary outcome**									
Coronary revascularisation									

Unexposed	834,643	4,602,759	9,211	1.00	(ref)	1.00	(ref)	1.00	(ref)
Exposed	208,122	1,251,337	2,887	1.11	1.05, 1.18	1.13	1.04, 1.24	1.08	0.98, 1.18

[1]Matched sets including one exposed patient and at least one unexposed patient.

[2]Estimated hazard ratios from Cox regression with current age as underlying timescale, stratified by matched set (matched on age at cohort entry, gender, date at cohort entry and practice).

Unadjusted: No adjustment.

Adjusted: Adjusted for current calendar period (1997-1999, 2000-2004, 2005-2009, 2010-2015), time since diagnosis (0-4, 5-9, 10-14, 15-19, 20+ years), IMD at cohort entry, and time-varying asthma.

Mediation model: Adjusted additionally for BMI and smoking at cohort entry, and time-varying hyperlipidaemia, hypertension, depression, anxiety, diabetes and severe alcohol use.

Table S9. Association between atopic eczema and cardiovascular outcomes, restricted to patients with at least one consultation with their GP in the year prior to cohort entry. Fitted to patients with complete data for all variables included in the models and from valid matched sets[1]. n = 1,586,719 (1,524,588 unique patients).

	n	P-Y at risk	Events	Hazard ratio and 99% confidence interval[2]					
				Unadjusted		Adjusted		Mediation model	
**Primary outcomes**									
Myocardial infarction									
Unexposed	1,225,744	7,492,477	14,250	1.00	(ref)	1.00	(ref)	1.00	(ref)
Exposed	360,975	2,384,676	5,277	1.08	1.03, 1.13	1.05	0.96, 1.14	1.05	0.96, 1.14
Unstable angina									
Unexposed	1,225,744	7,514,953	6,184	1.00	(ref)	1.00	(ref)	1.00	(ref)
Exposed	360,975	2,392,767	2,373	1.16	1.08, 1.25	1.19	1.05, 1.34	1.14	1.00, 1.30
Heart failure									
Unexposed	1,225,744	7,500,524	14,913	1.00	(ref)	1.00	(ref)	1.00	(ref)
Exposed	360,975	2,385,243	6,234	1.18	1.13, 1.24	1.15	1.06, 1.26	1.16	1.06, 1.26
Atrial fibrillation									
Unexposed	1,225,744	7,450,305	24,842	1.00	(ref)	1.00	(ref)	1.00	(ref)
Exposed	360,975	2,367,954	9,550	1.09	1.05, 1.13	1.08	1.01, 1.15	1.06	0.99, 1.13
Stroke									
Unexposed	1,225,744	7,490,153	18,378	1.00	(ref)	1.00	(ref)	1.00	(ref)
Exposed	360,975	2,383,783	6,869	1.07	1.02, 1.11	1.09	1.01, 1.18	1.08	0.99, 1.17
Cardiovascular death									
Unexposed	1,225,744	7,545,566	26,350	1.00	(ref)	1.00	(ref)	1.00	(ref)
Exposed	360,975	2,404,481	10,409	1.05	1.01, 1.09	0.96	0.89, 1.03	0.95	0.88, 1.02
**Secondary outcome**									

Coronary revascularisation									
Unexposed	1,225,744	7,488,558	13,368	1.00	(ref)	1.00	(ref)	1.00	(ref)
Exposed	360,975	2,383,086	4,814	1.07	1.02, 1.13	1.10	1.01, 1.21	1.07	0.98, 1.17

[1]Matched sets including one exposed patient and at least one unexposed patient.

[2]Estimated hazard ratios from Cox regression with current age as underlying timescale, stratified by matched set (matched on age at cohort entry, gender, date at cohort entry and practice).

Unadjusted: No adjustment.

Adjusted: Adjusted for current calendar period (1997-1999, 2000-2004, 2005-2009, 2010-2015), time since diagnosis (0-4, 5-9, 10-14, 15-19, 20+ years), IMD at cohort entry, and time-varying asthma.

Mediation model: Adjusted additionally for BMI and smoking at cohort entry, and time-varying hyperlipidaemia, hypertension, depression, anxiety, diabetes and severe alcohol use.

Table S10. Association between atopic eczema and cardiovascular outcomes, using alternative patient cohort[1]. Fitted to patients with complete data for all variables included in the models and from valid matched sets[2]. n = 1,937,732 (1,842,998 unique patients).

				Hazard ratio and 99% confidence interval[3]					
	n	P-Y at risk	Events	Unadjusted		Adjusted		Mediation model	
**Primary outcomes**									
Myocardial infarction									
Unexposed	1,547,230	9,434,873	17,239	1.00	(ref)	1.00	(ref)	1.00	(ref)
Exposed	390,502	2,557,480	5,585	1.10	1.05, 1.15	1.10	1.02, 1.20	1.07	0.99, 1.17
Unstable angina									
Unexposed	1,547,230	9,466,208	6,994	1.00	(ref)	1.00	(ref)	1.00	(ref)
Exposed	390,502	2,566,413	2,476	1.25	1.17, 1.33	1.34	1.19, 1.51	1.22	1.08, 1.38
Heart failure									
Unexposed	1,547,230	9,449,252	16,892	1.00	(ref)	1.00	(ref)	1.00	(ref)
Exposed	390,502	2,558,550	6,500	1.22	1.17, 1.28	1.20	1.10, 1.30	1.15	1.06, 1.26
Atrial fibrillation									
Unexposed	1,547,230	9,389,352	28,775	1.00	(ref)	1.00	(ref)	1.00	(ref)
Exposed	390,502	2,540,467	9,970	1.12	1.08, 1.16	1.08	1.01, 1.15	1.05	0.98, 1.12
Stroke									
Unexposed	1,547,230	9,434,858	21,391	1.00	(ref)	1.00	(ref)	1.00	(ref)
Exposed	390,502	2,556,917	7,195	1.08	1.03, 1.12	1.11	1.03, 1.20	1.08	1.00, 1.16
Cardiovascular death									
Unexposed	1,547,216	9,500,880	29,872	1.00	(ref)	1.00	(ref)	1.00	(ref)
Exposed	390,502	2,578,639	10,909	1.09	1.05, 1.13	1.00	0.94, 1.07	0.97	0.91, 1.04
**Secondary outcome**									
Coronary revascularisation									

Unexposed	1,547,230	9,432,504	16,074	1.00	(ref)	1.00	(ref)	1.00	(ref)
Exposed	390,502	2,556,150	5,085	1.15	1.10, 1.20	1.23	1.13, 1.34	1.16	1.07, 1.27

[1]Patients with an atopic eczema diagnosis but without two further treatments are included in the control pool for the entire duration of their follow-up. Patients in the exposed cohort (diagnosis and two treatments) are included as unexposed, up until their cohort entry (i.e. latest of their diagnosis and two treatments). The pool of exposed patients is the same as the main analysis above.

[2]Matched sets including one exposed patient and at least one unexposed patient.

[3]Estimated hazard ratios from Cox regression with current age as underlying timescale, stratified by matched set (matched on age at cohort entry, gender, date at cohort entry and practice).

Unadjusted: No adjustment.

Adjusted: Adjusted for current calendar period (1997-1999, 2000-2004, 2005-2009, 2010-2015), time since diagnosis (0-4, 5-9, 10-14, 15-19, 20+ years), IMD at cohort entry, and time-varying asthma.

Mediation model: Adjusted additionally for BMI and smoking at cohort entry, and time-varying hyperlipidaemia, hypertension, depression, anxiety, diabetes and severe alcohol use.

Table S11. Association between atopic eczema and cardiovascular outcomes, using alternative patient cohort[1]. Fitted to patients with complete data for all variables included in the models and from valid matched sets[2]. n = 2,348,708 2,247,330 unique patients).

				Hazard ratio and 99% confidence interval[3]					
	n	P-Y at risk	Events	Unadjusted		Adjusted		Mediation model	
**Primary outcomes**									
Myocardial infarction									
Unexposed	1,866,510	11,715,786	19,698	1.00	(ref)	1.00	(ref)	1.00	(ref)
Exposed	482,198	3,233,557	6,268	1.08	1.03, 1.12	1.04	0.96, 1.12	1.02	0.95, 1.11
Unstable angina									
Unexposed	1,866,510	11,752,460	8,317	1.00	(ref)	1.00	(ref)	1.00	(ref)
Exposed	482,198	3,244,130	2,795	1.18	1.10, 1.25	1.18	1.05, 1.32	1.12	1.00, 1.27
Heart failure									
Unexposed	1,866,510	11,734,024	19,160	1.00	(ref)	1.00	(ref)	1.00	(ref)
Exposed	482,198	3,236,228	6,920	1.12	1.08, 1.17	1.10	1.01, 1.19	1.08	0.99, 1.17
Atrial fibrillation									
Unexposed	1,866,510	11,666,794	32,055	1.00	(ref)	1.00	(ref)	1.00	(ref)
Exposed	482,198	3,214,738	10,920	1.09	1.05, 1.12	1.06	0.99, 1.13	1.03	0.97, 1.10
Stroke									
Unexposed	1,866,510	11,718,325	23,887	1.00	(ref)	1.00	(ref)	1.00	(ref)
Exposed	482,198	3,233,416	7,921	1.05	1.01, 1.09	1.07	0.99, 1.15	1.05	0.97, 1.13
Cardiovascular death									
Unexposed	1,866,491	11,794,738	33,169	1.00	(ref)	1.00	(ref)	1.00	(ref)
Exposed	482,197	3,258,280	11,513	1.01	0.98, 1.04	0.93	0.86, 0.99	0.89	0.83, 0.96
**Secondary outcome**									
Coronary revascularisation									

Unexposed	1,866,510	11,711,769	18,858	1.00	(ref)	1.00	(ref)	1.00	(ref)
Exposed	482,198	3,231,747	5,816	1.08	1.03, 1.13	1.12	1.03, 1.22	1.08	0.99, 1.17

[1]Exposed patients are atopic eczema diagnosis only (without the treatment criteria), and these patients are eligible to be a control up until their atopic eczema diagnosis.

[2]Matched sets including one exposed patient and at least one unexposed patient.

[3]Estimated hazard ratios from Cox regression with current age as underlying timescale, stratified by matched set (matched on age at cohort entry, gender, date at cohort entry and practice).

Unadjusted: No adjustment.

Adjusted: Adjusted for current calendar period (1997-1999, 2000-2004, 2005-2009, 2010-2015), time since diagnosis (0-4, 5-9, 10-14, 15-19, 20+ years), IMD at cohort entry, and time-varying asthma.

Mediation model: Adjusted additionally for BMI and smoking at cohort entry, and time-varying hyperlipidaemia, hypertension, depression, anxiety, diabetes and severe alcohol use.

Table S12. Summary statistics for covariates comparing patients with registrations from 2007 onwards with the overall cohort.

	Complete data for analysis variables & from valid matched set[1]			Registration 2007 onwards, complete data for analysis variables & from valid matched set[1]		
	Without atopic eczema n = 1,528,477 (79.8%)	With atopic eczema n = 387,439 (20.2%)	Total n = 1,915,916	Without atopic eczema n = 155,590 (75.2%)	With atopic eczema n = 51,242 (24.8%)	Total n = 206,832
**At entry to cohort**						
Gender						
Male	511,676 (33.5)	139,908 (36.1)	651,584 (34.0)	43,151 (27.7)	16,741 (32.7)	59,892 (29.0)
Female	1,016,801 (66.5)	247,531 (63.9)	1,264,332 (66.0)	112,439 (72.3)	34,501 (67.3)	146,940 (71.0)
Age (years)						
18-19	87,600 (5.7)	36,392 (9.4)	123,992 (6.5)	4,622 (3.0)	2,290 (4.5)	6,912 (3.3)
20-29	275,632 (18.0)	66,156 (17.1)	341,788 (17.8)	66,022 (42.4)	19,859 (38.8)	85,881 (41.5)
30-39	315,206 (20.6)	70,222 (18.1)	385,428 (20.1)	46,612 (30.0)	13,095 (25.6)	59,707 (28.9)
40-49	272,436 (17.8)	61,898 (16.0)	334,334 (17.5)	18,495 (11.9)	6,827 (13.3)	25,322 (12.2)
50-59	243,469 (15.9)	55,294 (14.3)	298,763 (15.6)	9,177 (5.9)	3,831 (7.5)	13,008 (6.3)
60-69	195,936 (12.8)	49,420 (12.8)	245,356 (12.8)	6,503 (4.2)	3,027 (5.9)	9,530 (4.6)
70-79	104,132 (6.8)	33,837 (8.7)	137,969 (7.2)	2,653 (1.7)	1,419 (2.8)	4,072 (2.0)
80+	34,066 (2.2)	14,220 (3.7)	48,286 (2.5)	1,506 (1.0)	894 (1.7)	2,400 (1.2)
IMD						
1 (Least deprived)	370,953 (24.3)	94,223 (24.3)	465,176 (24.3)	35,153 (22.6)	11,812 (23.1)	46,965 (22.7)
2	332,853 (21.8)	84,297 (21.8)	417,150 (21.8)	32,053 (20.6)	10,882 (21.2)	42,935 (20.8)
3	312,836 (20.5)	78,603 (20.3)	391,439 (20.4)	31,037 (19.9)	10,286 (20.1)	41,323 (20.0)
4	273,332 (17.9)	69,223 (17.9)	342,555 (17.9)	29,703 (19.1)	9,493 (18.5)	39,196 (19.0)
5 (Most deprived)	238,503 (15.6)	61,093 (15.8)	299,596 (15.6)	27,644 (17.8)	8,769 (17.1)	36,413 (17.6)
BMI						
Underweight	45,488 (3.0)	11,590 (3.0)	57,078 (3.0)	6,004 (3.9)	1,662 (3.2)	7,666 (3.7)
Normal weight	709,903 (46.4)	174,234 (45.0)	884,137 (46.1)	78,648 (50.5)	24,102 (47.0)	102,750 (49.7)
Overweight	484,434 (31.7)	123,352 (31.8)	607,786 (31.7)	42,850 (27.5)	14,802 (28.9)	57,652 (27.9)

Obese	288,652 (18.9)	78,263 (20.2)	366,915 (19.2)	28,088 (18.1)	10,676 (20.8)	38,764 (18.7)
Smoking						
Non-smoker	699,570 (45.8)	168,221 (43.4)	867,791 (45.3)	77,828 (50.0)	23,127 (45.1)	100,955 (48.8)
Current smoker	480,780 (31.5)	116,551 (30.1)	597,331 (31.2)	38,295 (24.6)	12,267 (23.9)	50,562 (24.4)
Ex-smoker	348,127 (22.8)	102,667 (26.5)	450,794 (23.5)	39,467 (25.4)	15,848 (30.9)	55,315 (26.7)
Diabetes	51,213 (3.4)	15,777 (4.1)	66,990 (3.5)	4,196 (2.7)	2,005 (3.9)	6,201 (3.0)
Hypertension	190,217 (12.4)	58,001 (15.0)	248,218 (13.0)	8,075 (5.2)	4,002 (7.8)	12,077 (5.8)
Hyperlipidaemia	59,376 (3.9)	19,342 (5.0)	78,718 (4.1)	3,404 (2.2)	1,868 (3.6)	5,272 (2.5)
Depression	300,699 (19.7)	95,131 (24.6)	395,830 (20.7)	33,454 (21.5)	14,788 (28.9)	48,242 (23.3)
Anxiety	194,289 (12.7)	65,248 (16.8)	259,537 (13.5)	20,503 (13.2)	9,456 (18.5)	29,959 (14.5)
Asthma	190,728 (12.5)	91,955 (23.7)	282,683 (14.8)	21,775 (14.0)	15,333 (29.9)	37,108 (17.9)
Severe alcohol use	28,803 (1.9)	8,730 (2.3)	37,533 (2.0)	5,525 (3.6)	2,274 (4.4)	7,799 (3.8)
**By exit from cohort**						
Diabetes	106,106 (6.9)	32,574 (8.4)	138,680 (7.2)	5,475 (3.5)	2,644 (5.2)	8,119 (3.9)
Hypertension	299,283 (19.6)	87,564 (22.6)	386,847 (20.2)	9,141 (5.9)	4,520 (8.8)	13,661 (6.6)
Hyperlipidaemia	123,187 (8.1)	37,652 (9.7)	160,839 (8.4)	4,130 (2.7)	2,221 (4.3)	6,351 (3.1)
Depression	426,864 (27.9)	133,394 (34.4)	560,258 (29.2)	38,950 (25.0)	17,007 (33.2)	55,957 (27.1)
Anxiety	301,356 (19.7)	98,410 (25.4)	399,766 (20.9)	25,342 (16.3)	11,449 (22.3)	36,791 (17.8)
Asthma	229,455 (15.0)	105,810 (27.3)	335,265 (17.5)	22,920 (14.7)	15,919 (31.1)	38,839 (18.8)
Severe alcohol use	48,121 (3.1)	14,337 (3.7)	62,458 (3.3)	6,365 (4.1)	2,649 (5.2)	9,014 (4.4)

[1]Matched sets including one exposed patient and at least one unexposed patient.

Table S13. Association between atopic eczema and cardiovascular outcomes, using only patients registered from 2007 onwards. Fitted to patients with complete data for all variables included in the models and from valid matched sets[1]. n = 206,832 (204,692 unique patients).

	n	P-Y at risk	Events	Hazard ratio and 99% confidence interval[2]					
				Unadjusted		Adjusted		Mediation model	
**Primary outcomes**									
Myocardial infarction									
Unexposed	155,590	299,151	153	1.00	(ref)	1.00	(ref)	1.00	(ref)
Exposed	51,242	109,663	91	1.17	0.74, 1.86	1.47	0.72, 3.00	1.44	0.66, 3.14
Unstable angina									
Unexposed	155,590	299,231	57	1.00	(ref)	1.00	(ref)	1.00	(ref)
Exposed	51,242	109,737	21	0.64	0.28, 1.46	0.55	0.15, 1.99	0.51	0.12, 2.22
Heart failure									
Unexposed	155,590	299,209	122	1.00	(ref)	1.00	(ref)	1.00	(ref)
Exposed	51,242	109,688	72	1.01	0.56, 1.82	1.03	0.41, 2.63	1.14	0.41, 3.17
Atrial fibrillation									
Unexposed	155,590	298,983	244	1.00	(ref)	1.00	(ref)	1.00	(ref)
Exposed	51,242	109,555	139	1.15	0.78, 1.68	1.15	0.65, 2.02	1.13	0.62, 2.07
Stroke									
Unexposed	155,590	299,105	197	1.00	(ref)	1.00	(ref)	1.00	(ref)
Exposed	51,242	109,635	127	1.70	1.12, 2.57	1.87	0.99, 3.55	1.85	0.92, 3.69
Cardiovascular death									
Unexposed	155,590	299,355	238	1.00	(ref)	1.00	(ref)	1.00	(ref)
Exposed	51,242	109,782	134	1.03	0.68, 1.56	0.84	0.45, 1.57	0.77	0.38, 1.56
**Secondary outcome**									
Coronary revascularisation									

Unexposed	155,590	299,028	203	1.00	(ref)	1.00	(ref)	1.00	(ref)
Exposed	51,242	109,623	103	1.04	0.70, 1.54	1.46	0.82, 2.60	1.46	0.79, 2.69

[1]Matched sets including one exposed patient and at least one unexposed patient.

[2]Estimated hazard ratios from Cox regression with current age as underlying timescale, stratified by matched set (matched on age at cohort entry, gender, date at cohort entry and practice).

Unadjusted: No adjustment.

Adjusted: Adjusted for current calendar period (1997-1999, 2000-2004, 2005-2009, 2010-2015), time since diagnosis (0-4, 5-9, 10-14, 15-19, 20+ years), IMD at cohort entry, and time-varying asthma.

Mediation model: Adjusted additionally for BMI and smoking at cohort entry, and time-varying hyperlipidaemia, hypertension, depression, anxiety, diabetes and severe alcohol use.

Table S14. Association between atopic eczema and cardiovascular outcomes, using only patients registered from 2007 onwards with complete ethnicity data. Fitted to patients with complete data for all variables included in the models and from valid matched sets[1]. n = 163,173 (161,470 unique patients).

				Hazard ratio and 99% confidence interval[2]									
						No adjustment for ethnicity				Adjusted for ethnicity			
	n	P-Y at risk	Events	Unadjusted		Adjusted		Mediation model		Adjusted		Mediation model	
**Primary outcomes**													
Myocardial infarction													
Unexposed	120,884	248,436	136	1.00	(ref)	1.00	(ref)	1.00	(ref)	1.00	(ref)	1.00	(ref)
Exposed	42,289	94,914	84	1.13	0.69, 1.84	1.41	0.65, 3.05	1.45	0.62, 3.42	1.62	0.77, 3.42	1.58	0.69, 3.61
Unstable angina													
Unexposed	120,884	248,496	52	1.00	(ref)	1.00	(ref)	1.00	(ref)	1.00	(ref)	1.00	(ref)
Exposed	42,289	94,973	21	0.68	0.30, 1.56	0.43	0.10, 1.80	0.32	0.05, 1.95	0.53	0.14, 1.99	0.49	0.11, 2.19
Heart failure													
Unexposed	120,884	248,489	103	1.00	(ref)	1.00	(ref)	1.00	(ref)	1.00	(ref)	1.00	(ref)
Exposed	42,289	94,936	63	0.88	0.47, 1.67	0.86	0.30, 2.44	0.92	0.29, 2.95	0.86	0.31, 2.37	0.94	0.31, 2.91
Atrial fibrillation													
Unexposed	120,884	248,293	209	1.00	(ref)	1.00	(ref)	1.00	(ref)	1.00	(ref)	1.00	(ref)
Exposed	42,289	94,824	119	1.07	0.71, 1.63	1.07	0.57, 2.02	0.99	0.50, 1.98	1.06	0.57, 1.99	1.00	0.51, 1.96
Stroke													
Unexposed	120,884	248,404	170	1.00	(ref)	1.00	(ref)	1.00	(ref)	1.00	(ref)	1.00	(ref)
Exposed	42,289	94,886	114	1.77	1.13, 2.77	1.88	0.91, 3.87	2.02	0.91, 4.48	1.94	0.97, 3.90	2.09	0.97, 4.48
Cardiovascular death													
Unexposed	120,884	248,610	184	1.00	(ref)	1.00	(ref)	1.00	(ref)	1.00	(ref)	1.00	(ref)
Exposed	42,289	95,017	108	0.94	0.59, 1.50	0.83	0.40, 1.71	0.69	0.29, 1.63	0.78	0.39, 1.57	0.62	0.27, 1.42
**Secondary outcome**													
Coronary revascularisation													
Unexposed	120,884	248,318	181	1.00	(ref)	1.00	(ref)	1.00	(ref)	1.00	(ref)	1.00	(ref)
Exposed	42,289	94,877	92	1.04	0.68, 1.58	1.54	0.82, 2.92	1.61	0.81, 3.20	1.57	0.84, 2.91	1.57	0.80, 3.06

[1]Matched sets including one exposed patient and at least one unexposed patient.

[2]Estimated hazard ratios from Cox regression with current age as underlying timescale, stratified by matched set (matched on age at cohort entry, gender, date at cohort entry and practice).

Unadjusted: No adjustment.

Adjusted: Adjusted for current calendar period (1997-1999, 2000-2004, 2005-2009, 2010-2015), time since diagnosis (0-4, 5-9, 10-14, 15-19, 20+ years), IMD at cohort entry, and time-varying asthma.

Mediation model: Adjusted additionally for BMI and smoking at cohort entry, and time-varying hyperlipidaemia, hypertension, depression, anxiety, diabetes and severe alcohol use.

Table S15. Association between atopic eczema and cardiovascular outcomes, including adjustment for high dose oral corticosteroid use. Fitted to patients with complete data for all variables included in the models and from valid matched sets1. n = 1,915,916 (1,842,759 unique patients).

	n	P-Y at risk	Events	Hazard ratio and 99% confidence interval[2] Mediation model	
**Primary outcomes**					
Myocardial infarction					
Unexposed	1,528,477	9,361,522	17,178	1.00	(ref)
Exposed	387,439	2,569,214	5,561	1.03	0.95, 1.12
Unstable angina					
Unexposed	1,528,477	9,392,370	7,059	1.00	(ref)
Exposed	387,439	2,578,165	2,460	1.16	1.03, 1.32
Heart failure					
Unexposed	1,528,477	9,375,383	16,983	1.00	(ref)
Exposed	387,439	2,570,412	6,441	1.16	1.06, 1.27
Atrial fibrillation					
Unexposed	1,528,477	9,316,331	28,571	1.00	(ref)
Exposed	387,439	2,552,311	9,892	1.07	1.00, 1.14
Stroke					
Unexposed	1,528,477	9,361,252	21,387	1.00	(ref)
Exposed	387,439	2,568,749	7,149	1.08	1.00, 1.16
Cardiovascular death					
Unexposed	1,528,477	9,427,420	30,116	1.00	(ref)
Exposed	387,439	2,590,305	10,813	0.96	0.89, 1.03
**Secondary outcome**					
Coronary revascularisation					
Unexposed	1,528,477	9,358,381	16,195	1.00	(ref)
Exposed	387,439	2,567,932	5,056	1.08	0.99, 1.18

[1]Matched sets including one exposed patient and at least one unexposed patient.

[2]Estimated hazard ratios from Cox regression with current age as underlying timescale, stratified by matched set (matched on age at cohort entry, gender, date at cohort entry and practice).

Mediation model: Adjusted for current calendar period (1997-1999, 2000-2004, 2005-2009, 2010-2015), time since diagnosis (0-4, 5-9, 10-14, 15-19, 20+ years), IMD at cohort entry, time-varying asthma, BMI and smoking at cohort entry, and time-varying hyperlipidaemia, hypertension, depression, anxiety, diabetes, severe alcohol use and high dose oral corticosteroid use.

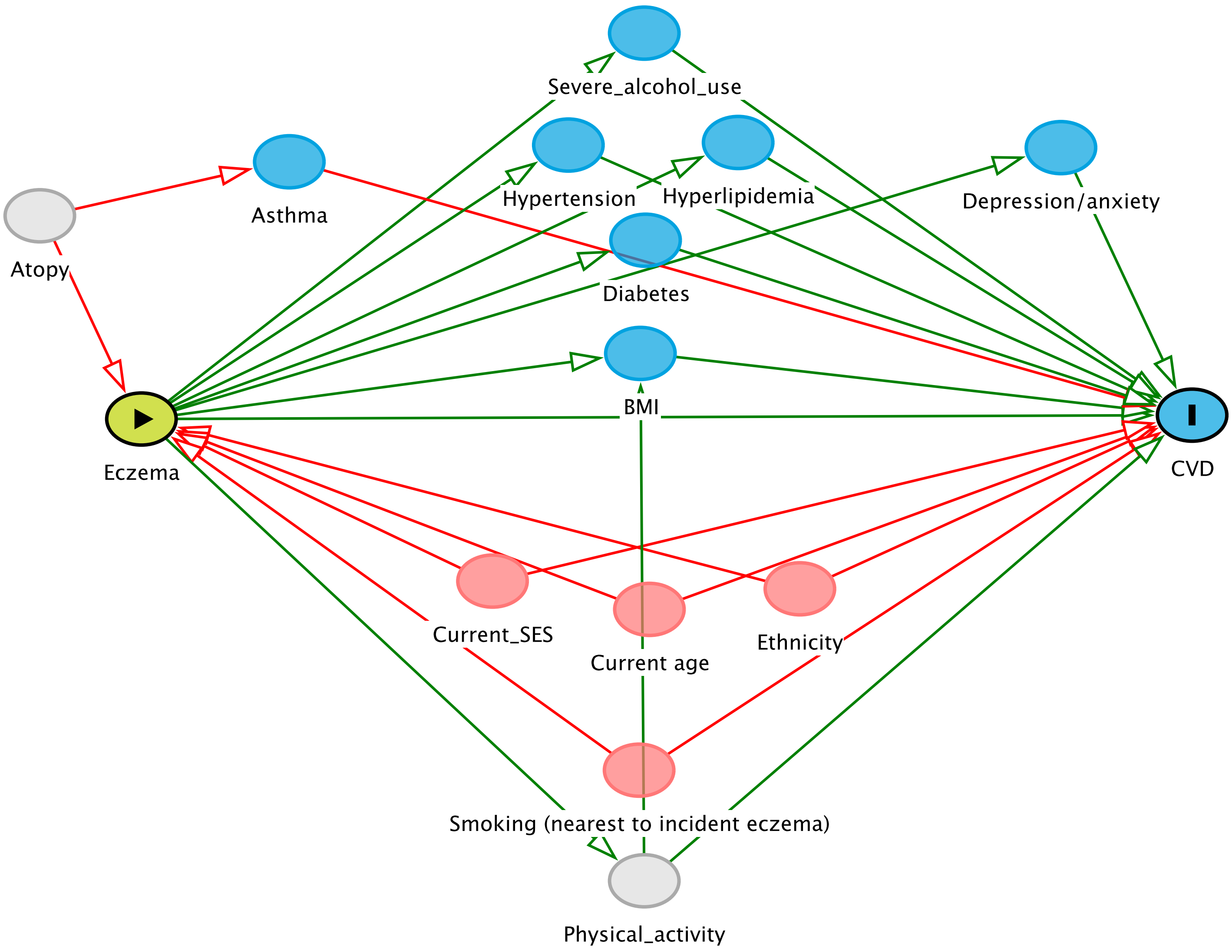
Severe_alcohol_use
Hypertension
Hyperlipidemia
Depression/anxiety
Asthma
Atopy
Diabetes
BMI
Eczema
CVD
Current_SES
Current age
Ethnicity
Smoking (nearest to incident eczema)
Physical_activity
